# Construction of machine learning-based models for screening the high-risk patients with gastric precancerous lesions

**DOI:** 10.1186/s13020-025-01059-4

**Published:** 2025-01-07

**Authors:** Shuxian Yu, Haiyang Jiang, Jing Xia, Jie Gu, Mengting Chen, Yan Wang, Xiaohong Zhao, Zehua Liao, Puhua Zeng, Tian Xie, Xinbing Sui

**Affiliations:** 1 School of Pharmacy, Hangzhou Normal University, Hangzhou, China; 2 Department of Medical Oncology, The Affiliated Hospital of Hangzhou Normal University, Hangzhou, China; 3 The First Affiliated Hospital of Zhejiang Chinese Medicine University, Hangzhou, China; 4 The Affiliated Hospital of Hunan Academy of Traditional Chinese Medicine, Changsha, Hunan China

**Keywords:** Precancerous lesions of gastric cancer, Gastric cancer, Machine learning, Deep learning, Traditional Chinese medicine

## Abstract

**Background:**

The individualized prediction and discrimination of precancerous lesions of gastric cancer (PLGC) is critical for the early prevention of gastric cancer (GC). However, accurate non-invasive methods for distinguishing between PLGC and GC are currently lacking. This study therefore aimed to develop a risk prediction model by machine learning and deep learning techniques to aid the early diagnosis of GC.

**Methods:**

In this study, a total of 2229 subjects were recruited from nine tertiary hospitals between October 2022 and November 2023. We designed a comprehensive questionnaire, identified statistically significant factors, and created a web-based column chart. Then, a risk prediction model was subsequently developed by machine learning techniques. In addition, a tongue image-based risk prediction model was established by deep learning algorithms.

**Results:**

Based on logistic regression analysis, a dynamic web-based nomogram was developed and it is freely accessible at: https://yz6677.shinyapps.io/GC67/. Then, the prediction model was established using ten different machine learning algorithms and the Random Forest (RF) model achieved the highest accuracy at 85.65%. According with the predictive results, the top 10 key risk factors were age, traditional Chinese medicine (TCM) constitution type, tongue coating color, tongue color, irregular meals, pickled food, greasy fur, over-hot eating habit, anxiety and sleep onset latency. These factors are all significant risk indicators for the progression of PLGC patients to GC patients. Subsequently, the Swin Transformer architecture was used to develop a tongue image-based model for predicting the risk for progression of PLGC. The verification set showed an accuracy of 73.33% and an area under curve (AUC) greater than 0.8 across all models.

**Conclusions:**

Our study developed machine learning and deep learning-based models for predicting the risk for progression of PLGC to GC, which will offer the assistance to determine the high-risk patients from PLGC and improve the early diagnosis of GC.

**Graphical Abstract:**

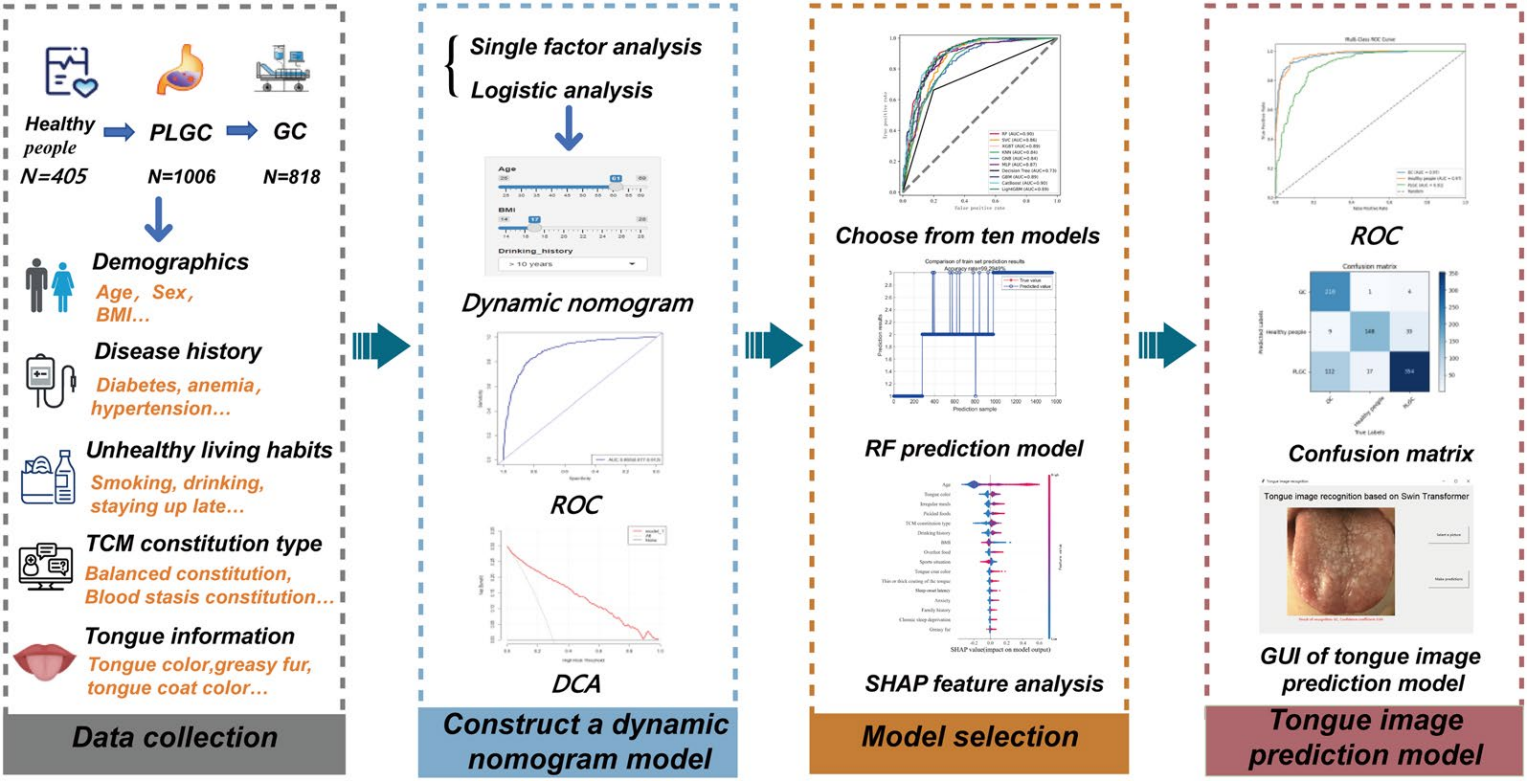

**Supplementary Information:**

The online version contains supplementary material available at 10.1186/s13020-025-01059-4.

## Background

Currently, the incidence and mortality of gastric cancer (GC) in China remain high and more than 80% of patients are diagnosed at an advanced stage [1]. Therefore, identifying the high-risk populations from precancerous lesions of gastric cancer (PLGC) is crucial for reducing GC incidence. PLGC is primarily characterized by intestinal metaplasia and dysplasia of the gastric mucosa [2, 3].

Disease risk factors are rarely singular; instead, multiple factors typically contribute to a state of compound exposure. For instance, unhealthy lifestyles, including poor diet and lack of exercise, negatively impact health, and correcting such habits can lower the risk of GC. Furthermore, traditional Chinese medicine (TCM) constitution theory is applied to explore the characteristics, evolution patterns, influencing factors, and classification criteria of various constitutions [4]. This framework can thus be used to guide the prevention, diagnosis, treatment, rehabilitation, and overall health management. Identifying patients with a higher risk of developing GC and adjusting their TCM constitution in advance will facilitate the early prevention of GC. Tongue images are also closely associated with gastric diseases [5]. So, we collected the tongue image data from all the subjects to make a more efficient and accurate assessment of progression of PLGC. However, these factors should not be examined in isolation; instead, their combined effects must be comprehensively analyzed. Due to variable collinearity and high-dimensional data, traditional epidemiological methods are insufficient for analyzing this complexity. Random Forest (RF) analysis can account for interactions and nonlinear correlations among multiple factors simultaneously, identifying key variables and making it well-suited for high-dimensional data [6]. Additionally, the nomogram, a widely-used visual prediction tool in oncology, facilitates clinicians’ ability to predict prognosis and plays a significant role in promoting personalized medicine [7, 8].

Moreover, the Transformer architecture, recently published in Natural Language Processing (NLP), has demonstrated exceptional recognition accuracy in Computer Vision (CV) tasks, along with strong image classification capabilities. The Swin Transformer [9], an enhancement of the original Transformer model, enables global attention operations on large-scale images through a self-attention mechanism based on moving windows, which is particularly suited for processing remote sensing data. The moving window mechanism within the self-attention framework facilitates global modeling capabilities. For feature extraction, a hierarchical construction approach, similar to that of Convolutional Neural Networks (CNN) [10], is employed. This method reduces the image size to filter out less significant features and redundant information, while retaining essential data. The Swin Transformer architecture is particularly well-suited for processing large 2D or higher-dimensional image datasets. Since its introduction, this model has been widely adopted and praised within the academic community.

In this study, univariate analysis, binary logistic regression analysis, random forest model, Shapley Additive exPlanations (SHAP) feature analysis, and dynamic nomogram were used to comprehensively identify the important risk factors for progression of PLGC to GC patients. In addition, Swin Transformer model is used to analyze and predict GC through tongue images, providing more new clues for the prevention of GC.

## Methods

### Research object

Between October 2022 and January 2024, patient information was collected from nine tertiary or above hospitals, including The Affiliated Hospital of Hangzhou Normal University, the Second Affiliated Hospital of Zhejiang University, Zhejiang Cancer Hospital, the Affiliated Hospital of Hunan Academy of Traditional Chinese Medicine, Tianjin Cancer Hospital, Tianjin Nankai Hospital, Tianjin Second People’s Hospital, Chongqing Cancer Hospital, and Chongqing Hospital of Traditional Chinese Medicine. Based on pathological findings, 1006 patients diagnosed with atrophic gastritis via gastroscopy and intestinal metaplasia via gastroscopy were included in the study. In total, 818 GC patients meeting the “People’s Republic of China Health Industry Standard: GC Diagnostic Criteria (WS316-2010)” were recruited. Additionally, 405 healthy individuals were recruited.

Inclusion criteria were as follows: (i) Age 18–75 years; (ii) Clear consciousness, ability to communicate effectively with investigators, and capacity to read and express language clearly; (iii) Willingness to complete questionnaires and provide informed consent.

Exclusion criteria included: (i) Withdrawal during the study; (ii) Presence of severe comorbidities affecting the heart, cerebrovascular system, liver, kidney, hematopoietic system, or other serious organic diseases; (iii) Individuals with mental illness, intellectual disabilities, or language impairments.

### Investigation methods and contents

Drawing upon similar research methods both domestically and internationally, we designed the questionnaire. The questionnaire covered the following areas: (i) Demographic information, including gender, age, height, and body mass index (BMI); (ii) Unhealthy lifestyle habits, including smoking and alcohol drinking history; (iii) Medical history, including hypertension, diabetes, anemia, and family history; (iv) Sleep conditions, including sleep onset latency, insomnia, irregular sleep, staying up late, somnolence, and chronic sleep deprivation; (v) Social and psychological factors, including tension, depression, anxiety, and high work-related stress; (vi) Physical activity; (vii) Dietary habits, such as preferences for cold, pickled, spicy, greasy, and fast foods, as well as a high-salt diet, excessively hot food, irregular meals, and smoked foods; (viii) Tongue image data, including tongue color, coating (thin or thick), greasy or curdy fur, peeling, plumpness or slenderness, and local features; (ix) TCM constitution type, including balanced, Qi-deficiency, Yang-deficiency, Yin-deficiency, phlegm-dampness, damp-heat, blood stasis, Qi stagnation, and inherited special constitutions.

The questionnaire survey was administered by trained medical personnel with standardized procedures. Double-entry data input was performed by trained staff, and all data were reviewed prior to entry to ensure accuracy and quality. The collected questionnaire data were assigned values (Table 1).

**Table 1:** Assignment of questionnaire variables

Variable	Assignment(score)
Gender	Male = 0, Female = 1
Age (years)	≤ 30 = 0, 31–40 = 1, 41–50 = 2, > 50 = 3
BMI	< 18.5 = 0, 18.5–24 = 1, > 24 = 2
Alcohol drinking history	No = 0, < 5 years = 1, 5–10 years = 2, > 10 years = 3
Smoking history	No = 0, < 5 years = 1, 5–10 years = 2, > 10 years = 3
Family history	No = 0, Yes = 1
Hypertension	No = 0, Yes = 1
Diabetes	No = 0, Yes = 1
Anaemia	No = 0, Yes = 1
Sleep onset latency	< 30 min = 0, 30-60 min = 1, > 60 min = 2
Insomnia	No = 0, < three months = 1, ≥ three months = 2
Irregular sleep	No = 0, Yes = 1
Stay up late	< 11 p.m. = 0, 11 p.m.—12 a.m. = 1, 12 a.m.—1 a.m. = 2, > 1 a.m. = 3
Somnolence	No = 0, Yes = 1
Chronic sleep deprivation	No = 0, Yes = 1
Tension	Never = 0, Minor = 1, Severe = 2
Anxiety	Never = 0, Minor = 1, Severe = 2
Depression	Never = 0, Minor = 1, Severe = 2
High work pressure	Never = 0, Minor = 1, Severe = 2
Sports situation	Less = 0, Everyday = 1, Every 3–5 days = 2, Once a week = 3
Cold food	Never = 0, Sometimes = 1, Often = 2
Pickled food	Never = 0, Sometimes = 1, Often = 2
Spicy food	Never = 0, Sometimes = 1, Often = 2
Fast eating	Never = 0, Sometimes = 1, Often = 2
Greasy food	Never = 0, Sometimes = 1, Often = 2
High-salt diet	No = 0, Yes = 1
Overhot food	Never = 0, Sometimes = 1, Often = 2
Irregular meals	Never = 0, Sometimes = 1, Often = 2
Smoked food	Never = 0, Sometimes = 1, Often = 2
Tongue color	Light red tongue = 0, Pale tongue = 1, Crimson tongue = 2, Livid tongue = 3
Tongue coating color	White tongue fur = 0, Yellow tongue fur = 1, Grey tongue fur = 2, Black-tongue fur = 3
Thin or thick coating of the tongue	Thin fur = 0, Thick fur = 1
Greasy fur	No = 0, Yes = 1
Curdy fur	No = 0, Yes = 1
Peeling tongue fur	No = 0, Yes = 1
Plumpness and slenderness of tongue	Slender tongue = 0, Bulgy tongue = 1
Tongue local features	Normal = 0, Teeth-printed tongue = 1, Spotted tongue = 2, Cracked tongue = 3
Abnormal condition of the tongue	Normal = 0, The edge of the tongue is red = 1, The tongue has petechiae or ecchymosis = 2
TCM constitution type	Balanced_constitution = 0, Qi-deficiency constitution = 1, Yang-deficiency constitution = 2, Yin-deficiency constitution = 3, Phlegm-dampness constitution = 4, Dampness-heat constitution = 5, Blood stasis constitution = 6, Qi stagnation constitution = 7, Inherited special constitution = 8

### Statistical method

The χ^2^ test was conducted using GraphPad Prism software. Risk factors with statistical significance (*P* < 0.05) were further analyzed using a binary logistic regression model in SPSS 27.0 software. Statistically significant factors (*P* < 0.05) identified through logistic regression were then used to establish the data set. The dataset was split with 70% used for training and 30% for testing. A traditional nomogram was constructed using lrm, rms, DynNom, and other packages in R 4.3.0 to create a network-based dynamic nomogram.

### Establishment of prediction model based on ML

Support Vector Classification (SVC), K-nearest Neighbor (KNN), RF, Extreme Gradient Boosting (XGBoost), Gaussian Naive Bayes (GNB), Multilayer Perceptron (MLP), Decision Tree, Gradient Boosting Machine (GBM), Category Boosting (CatBoost), Light Gradient Boosting Machine (LightGBM), and other machine learning (ML) methods were used to construct prediction models and compared for their predictive accuracy. The experiments were conducted in a Jupyter Notebook environment. The RF prediction model was implemented using Matlab. SHAP feature analysis was performed to interpret the model's predictions.

### Establishment of Tongue Image GC Prediction Model Based on Deep Learning and GUI Development

The size of each image in the original data of tongue image is not uniform. This experiment resamples all images to adjust the size of each image to 224 × 224. In addition, in order to increase the stability of the model, this experiment, like other mainstream computer vision model train processes, uses data enhancement operations such as clipping, flipping, and normalization. The algorithm in this paper mainly relies on PyTorch, the mainstream open source framework of deep learning, and the software uses PyCharm-2022.3.3. Description of the hyperparameters used in the experiment: the Class number is 3, the Train Epochs is 50, the Batch size is 32, and the Learning rate is 0.0001. A Graphic User Interface (GUI) was created using the Tkinter module from the Python standard library.

## Results

### Single factor analysis of individual basic situation

The study enrolled 2229 participants, including 405 healthy people, 1006 PLGC patients, and 818 GC patients. Pairwise single factor analysis was performed between these three groups. The results of single factor analysis of healthy people and PLGC patients, healthy people and GC patients were shown in the supplementary materials.

Among the results, age and BMI were found to be significantly different between healthy people and PLGC patients, between healthy people and GC patients, and between PLGC and GC patients (*P* < 0.05) (Table S1–3). However, there was no significant difference in gender between above the groups (*P* > 0.05) (Table S1–3). In the further study, age (> 50 years) and BMI (< 18.5) were determined as the risk factors for PLGC, GC, and the progression of PLGC to GC.

### Univariate analysis of drinking and smoking history

There were significant differences in alcohol drinking history between healthy people and PLGC patients, between healthy people and GC patients, and between PLGC and GC patients (*P* < 0.05) (Table S4–6). However, there were no significant differences in smoking history between above the groups (*P* > 0.05) (Table S4–6). These results suggested that alcohol drinking history was associated with increased risk of PLGC, GC, and the progression of PLGC to GC.

### Single factor analysis of disease history

There were remarkable differences in family history between healthy people and PLGC patients, between healthy people and GC patients, and between PLGC and GC patients (*P* < 0.05). There were no significant differences in hypertension, diabetes, and anemia between healthy people and PLGC patients and between PLGC patients and GC patients (*P* > 0.05). However, there was a statistical difference in anemia between healthy people and GC patients (*P* < 0.05) (Table S7–9). So, family history was an important risk factor for PLGC, GC, and the progression of PLGC to GC.

### Univariate analysis of sleep status

The sleep conditions are divided into: sleep onset latency (minor, less than 30 min to fall asleep; medium, 30–60 min to fall asleep; severe, more than 60 min to fall asleep), insomnia (It takes less than 5 h to fall asleep, and has difficulty falling asleep and waking up early and other conditions). According to the duration of the disease, insomnia is divided into short-term insomnia (< 3 months) and chronic insomnia (≥ 3 months). Falling asleep after 11 PM is considered to be staying up late, and there are four levels based on different times. Irregular sleep times, poor sleep quality, or too much sleep and not enough sleep are considered irregular sleep. Sleep less than 7 h a day and long-term existence of this situation is considered to be long-term sleep deprivation. As a result, there were statistical differences in irregular sleep between healthy people and PLGC patients (*P* < 0.05), while there were no remarkable differences in sleep conditions such as sleep onset latency, insomnia, stay up late, somnolence and chronic sleep deprivation between healthy people and PLGC patients (*P* > 0.05) (Table S10). There were statistical differences in sleep conditions such as sleep onset latency, irregular sleep and chronic sleep deprivation between healthy people and GC patients (*P* < 0.05), while there were no statistical differences in insomnia, stay up late and lethargy between the two groups (*P* > 0.05) (Table S11). There were significant differences in sleep conditions such as sleep onset latency and chronic sleep deprivation between PLGC and GC patients (*P* < 0.05), while there were no statistical differences in sleep conditions such as insomnia, irregular sleep, staying up late and somnolence between PLGC and GC patients (*P* > 0.05) (Table S12). Taken together, our results showed that sleep onset latency and chronic sleep deprivation were important risk factors for the progression of PLGC to GC.

### Single factor analysis of psychosocial factors

According to the results of single factor analysis, there was no significant difference in social psychological factors such as tension, anxiety, depression, and high work pressure between healthy people and PLGC patients (*P* > 0.05) (Table S13). A significant difference in anxiety was observed between healthy people and GC patients, as well as between PLGC and GC patients (*P* < 0.05) (Table S14-15). However, there was no significant difference in stress, depression and work pressure between above two groups (*P* > 0.05). These results indicated that individuals who were prone to anxiety were more likely to progress from PLGC to GC.

### Univariate analysis of sports situation

There were statistically significant differences in exercise status between healthy people and PLGC patients, between healthy people and GC patients, and between PLGC and GC patients (*P* < 0.05) (Table S16–18). So, exercising once a week or every 3–5 days, rather than daily, decreased the risk of PLGC and GC, as well as the progression from PLGC to GC.

### Univariate analysis of diet

The significant differences (*P* < 0.05) were observed in dietary factors such as pickled food, overhot food consumption, and irregular meals between the healthy population and patients with PLGC, between the healthy population and GC patients, as well as between PLGC and GC patients (Table S19–21). However, the other factors such as excessive cold food consumption, preference for greasy food, spicy food, fast eating, high salt intake, and smoked food did not show significant differences (*P* > 0.05) between the healthy population and PLGC patients, between the healthy population and GC patients, or between PLGC and GC patients (Table S19–21). Therefore, the consumption of pickled food, overhot food consumption, and irregular meals is increased risk of both PLGC, GC, and the progression of PLGC to GC.

### Single factor analysis of tongue image information

There were significant differences between healthy people and PLGC patients in tongue color (pale tongue, crimson tongue, and livid tongue), tongue coating color (yellow tongue fur, grey tongue fur, and black-tongue fur), thick tongue coating, greasy fur, and curdy fur (*P* < 0.05) (Table S22). In terms of tongue color (pale tongue, crimson tongue, and livid tongue), tongue coating color (yellow tongue fur, grey tongue fur, and black-tongue fur), thick tongue coating, and greasy fur, there were significant differences between healthy people and GC patients (*P* < 0.05) (Table S23). Significant differences were observed in tongue color (pale tongue, crimson tongue, and livid tongue), tongue coating color (yellow tongue fur, grey tongue fur, and black tongue fur), thick tongue coating, and greasy fur between PLGC and GC patients (*P* < 0.05). No significant differences were found in curdy fur, peeling fur, plumpness and slenderness of tongue, tongue local features and abnormal condition of the tongue between the two groups (*P* > 0.05) (Table S24). These results indicated that abnormal tongue color, tongue coating color, thick tongue coating, and greasy fur were risk factors for PLGC, GC, and the progression of PLGC to GC.

### Single factor analysis of TCM constitution types

There were remarkable differences in TCM constitution type between healthy people and PLGC patients, between healthy people and GC patients, and between PLGC and GC patients (*P* < 0.05). Further studies revealed that yang-deficiency constitution, phlegm-dampness constitution, and damp-heat constitution were same risk factors for PLGC, GC, and the progression from PLGC to GC. (Table S25–27).

### Binary logistic regression analysis of basic information, lifestyle, and TCM constitution type of the study subjects

The variables with statistical significance in the univariate analysis were used as explanatory variables for binary Logistic regression analysis. Age: 31–40 (OR = 18.172), 41–50 (OR = 56.469), > 50 (OR = 38.525), frequent consumption of pickled food (OR = 4.398), pale tongue (OR = 2.832), crimson tongue (OR = 4.127), yellow tongue fur (OR = 2.087), thick tongue coating (OR = 1.621), TCM constitution type Qi-deficiency constitution (OR = 9.964), Yang-deficiency constitution (OR = 7.293), phlegm-dampness constitution (OR = 11.981), damp-heat constitution (OR = 8.109), Qi stagnation constitution (OR = 20.997), and inherited special constitution (OR = 8.468) were risk factors for PLGC in healthy people. Whereas, BMI of 18.5 to 24.0 (OR = 0.179), exercise daily (OR = 0.495), exercise once every 3 to 5 days (OR = 0.412), and exercise once a week (OR = 0.395) were protective factors against PLGC in healthy people (Table S28).

Statistically significant variables selected by one-way analysis were used as explanatory variables to analyze the binary Logistic regression model between healthy people and GC patients. The results showed that age: 31–40 years (OR = 73.942), 41–50 years (OR = 38.989), > 50 years (OR = 229.527), more than 10 years of alcohol drinking history (OR = 16.571), family history (OR = 6.302), sleep onset latency of 30–60 min (OR = 5.639), chronic sleep deficiency (OR = 7.189), minor anxiety (OR = 6.387), frequent consumption of pickled food (OR = 27.353), frequent consumption of overhot food (OR = 10.227), occasional irregular meals (OR = 16.009), pale tongue color (OR = 5.952), crimson tongue (OR = 13.698), yellow tongue fur (OR = 7.472), thick tongue coating (OR = 4.180), TCM constitution type Qi-deficiency constitution (OR = 9.300), Yang deficiency constitution (OR = 78.785), Phlegm-dampness constitution (OR = 134.360), Dampness-heat constitution (OR = 23.743), Qi stagnation constitution (OR = 131.360) and inherited special constitution (OR = 83.093) were the risk factors for GC in healthy people. Whereas, BMI of 18.5 to 24.0 (OR = 0.040) was protective factors against GC in healthy people (Table S29).

Binary Logistic regression model analysis was performed on PLGC patients and GC patients with statistically significant variables selected by univariate analysis as explanatory variables. And the results showed that age 41 – 50 years old (OR = 5.995) > 50 years old (OR = 48.691), alcohol drinking history (less than 5 years old, OR = 2.406; 5 – 10 years, OR = 1.736; more than 10 years, OR = 2.339), family history (OR = 1.409), sleep onset latency of 30–60 min (OR = 1.528), chronic sleep deprivation (OR = 2.032), minor anxiety (OR = 1.762), exercise once every 3 to 5 days (OR = 0.485), exercise once a week (OR = 0.461), frequent consumption of pickled food (OR = 2.519), frequent overhot food (OR = 2.549), pale tongue (OR = 2.219), crimson tongue (OR = 2.252), livid tongue (OR = 1.811), grey tongue fur (OR = 1.905), black tongue fur (OR = 4.314), thick tongue coating (OR = 1.381), greasy fur (OR = 1.461), Yang-deficiency constitution (OR = 2.550), phlegm-dampness constitution (OR = 2.374), dampness-heat constitution (OR = 2.535) are the risk factors of development of PLGC into GC. BMI of 18.5 to 24.0 (OR = 0.485) and exercise once every 3 to 5 days (OR = 0.485) per week (OR = 0.461) were protective factors against progression of PLGC to GC (Table S30).

### Construction and verification of risk prediction nomogram model between PLGC patients and GC patients

**Fig. 1:**
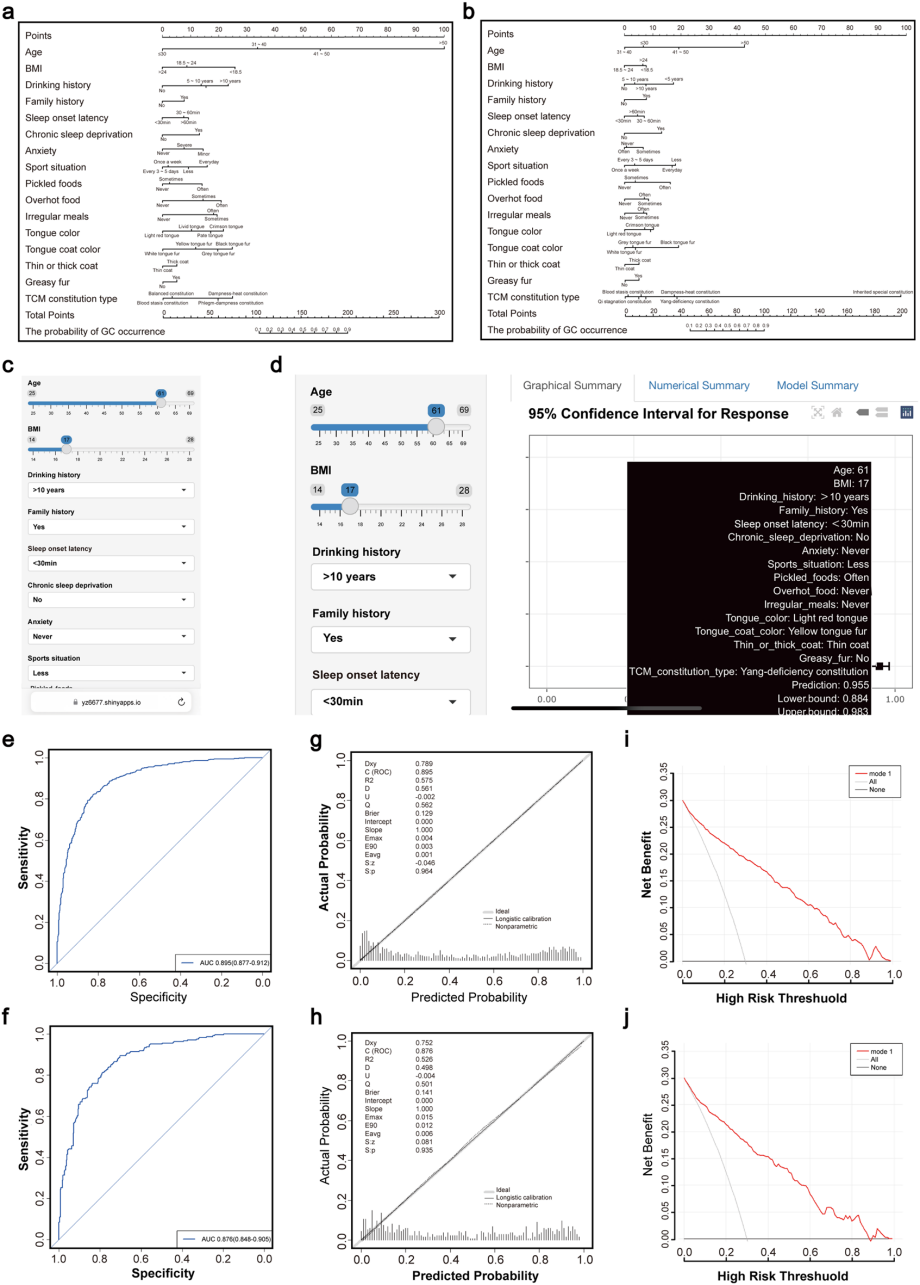
Web-based dynamic nomogram model of risk prediction between PLGC patients and GC patients. **a** A nomogram of the train set. **b** A nomogram of the validation set. **c** Web-based dynamic nomogram. **d** The probability of GC in a PLGC patient was predicted by a dynamic nomogram. **e** The AUC of the train set predicted by the dynamic nomogram was 0.895. **f** The AUC of the validation set is 0.876. **g** Calibration curve of the train set. **h** Calibration curve of the test set; **i** dca analysis diagram of the train se. **j** dca analysis diagram of validation set

The risk factors that were statistically significant (*P* < 0.05) from the logistic regression analysis were selected to establish the dataset. The dataset was split in a 7:3 ratio, with 70% used as the training set and 30% as the test set. A risk prediction nomogram model for PLGC and GC patients was constructed using R version 4.3.0, as shown in Fig. 1. Based on the data, we created a column diagram for the training set (Fig. 1A) and a column diagram for the testing set (Fig. 1B). A dynamic nomogram was developed in Fig. 1C, accessible via the web platform (https://yz6677.shinyapps.io/GC67/), which could make the visualization and personalized risk prediction of GC in PLGC patients.

Here is a case of a 61-year-old PLGC patient with a BMI of 17, a 10-year history of alcohol consumption, a family history of the disease, no regular exercise, frequent consumption of pickled food, irregular meals, yellow and greasy tongue fur, and Yang-deficiency constitution. According to the predictive model, the likelihood of this patient developing GC was estimated to be 95.5% (88.4%, 98.3%) (Fig. 1D).

The area under curve (AUC) of the predictive model for the training set based on the dynamic nomogram was 0.895 (95% CI 0.877–0.912) (Fig. 1E), while the AUC for the validation set was 0.876 (95% CI 0.848–0.905) (Fig. 1F). The maximum deviation between the dynamic nomogram constructed in this study and the ideal curve was 0.004 for the training set and 0.015 for the test set (Fig. 1G). The minimum deviation between the model and the ideal curve was 0.001 for the training set and 0.006 for the test set (Fig. 1H). The height of the nomogram curves for both the training and test sets was very close to that of the ideal curve, indicated good model performance. Decision curve analysis demonstrated that PLGC patients could clinically benefit from using this model to predict GC risk, suggested that the predictive model had good clinical utility (Fig. 1I, J).

### Comparison of ten ML methods

**Fig. 2:**
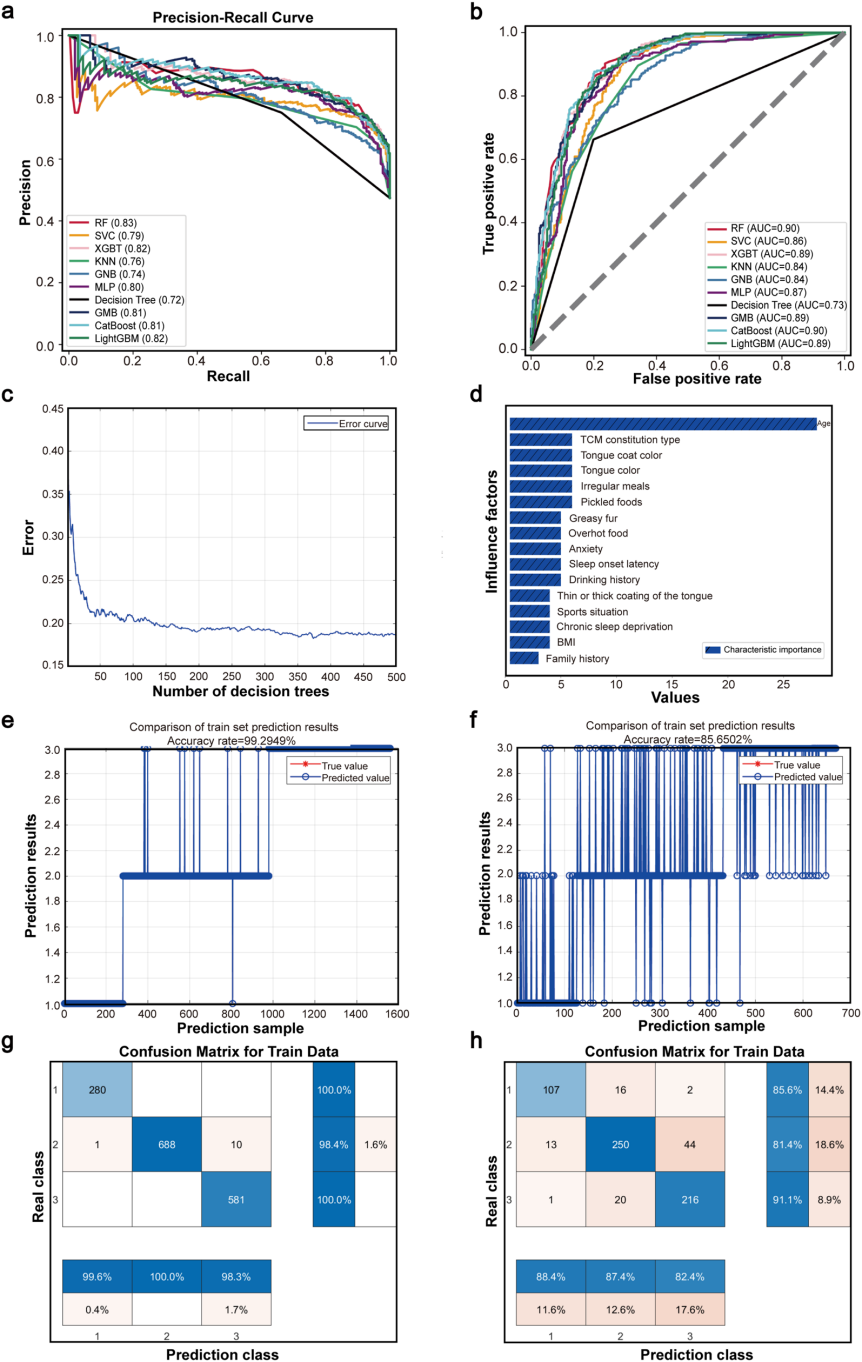
Ten ML methods were compared and the predictive model was built by RF. **a** Ten ML methods are used to compare the accuracy of the model. **b** ROC diagram of model comparison between ten ML methods. **c** Error curve. **d** Ranking map of important features. **e** The accuracy of the train set was 99.29%. **f** The accuracy of test set was 85.65%. **g** Confusion matrix of the train set. **h** Confusion matrix of test set

According to the results of the multi-factor analysis, statistically significant characteristics were selected. RF, SVC, KNN, XGBoost, GNB, MLP, Decision Tree, GBM, CatBoost, and LightGBM machine learning algorithms were applied to the pre-processed datasets, and their performance scores were evaluated. The results indicated that the RF model achieved the highest accuracy at 0.83 (Table 2). The recall rates of these ten machine learning algorithms indicated that the RF algorithm achieved the highest accuracy (Fig. 2A). Analysis of the receiver operating characteristic (ROC) curves of models trained with various machine learning methods revealed that both RF and CatBoost exhibited the highest AUC values (Fig. 2B). However, a comparison of the accuracy results in Fig. 2A showed that RF outperformed CatBoost was the primary model for subsequent experiments.

**Table 2:** Accuracy evaluation table of ten ML models

Model type	Category	Precision	Recall	F1-score	Accuracy
RF	Healthy peopl	0.85	0.81	0.83	0.83
	PLGC	0.81	0.84	0.82	
	GC	0.85	0.82	0.83	
SVC	Healthy peopl	0.86	0.68	0.76	0.79
	PLGC	0.75	0.86	0.80	
	GC	0.83	0.74	0.78	
XGBoost	Healthy peopl	0.81	0.79	0.8	0.82
	PLGC	0.80	0.83	0.81	
	GC	0.85	0.83	0.84	
KNN	Healthy peopl	0.71	0.72	0.72	0.76
	PLGC	0.74	0.78	0.76	
	GC	0.81	0.75	0.78	
GNB	Healthy peopl	0.61	0.78	0.69	0.74
	PLGC	0.78	0.64	0.71	
	GC	0.78	0.86	0.81	
MLP	Healthy peopl	0.79	0.77	0.78	0.80
	PLGC	0.80	0.78	0.79	
	GC	0.80	0.84	0.82	
Decision tree	Healthy peopl	0.66	0.79	0.72	0.71
	PLGC	0.73	0.64	0.68	
	GC	0.72	0.76	0.74	
GBM	Healthy peopl	0.81	0.79	0.80	0.81
	PLGC	0.79	0.81	0.8	
	GC	0.83	0.82	0.82	
CatBoost	Healthy peopl	0.82	0.80	0.81	0.81
	PLGC	0.80	0.80	0.80	
	GC	0.82	0.83	0.82	
LightGBM	Healthy peopl	0.82	0.80	0.81	0.82
	PLGC	0.81	0.82	0.81	
	GC	0.84	0.84	0.84

The prediction model based on RF was implemented in Matlab, with the training and test sets divided in a 7:3 ratio. The model’s average error was minimized when the number of decision trees was set to 500 and the minimum number of leaves was 1, as shown by its error curve (Fig. 2C). According to the ranking of important features for risk prediction between PLGC and GC patients, the top 10 key risk factors were age, TCM constitution type (Yang-deficiency constitution, Phlegm-dampness constitution and Dampness-heat constitution), tongue coating color, tongue color, irregular meals, consumption of pickled food, greasy tongue coating, overhot eating habits, anxiety, and sleep onset latency (Fig. 2D). Among TCM constitution types, Yang-deficiency constitution, Phlegm-dampness constitution, and Dampness-heat constitution were the main risk factors for gastric cancer (Table S30). The training set achieved an accuracy of 99.29% (Fig. 2E), while the testing set reached an accuracy of 85.65% (Fig. 2F). The confusion matrix of the training set (Fig. 2G) and the testing set (Fig. 2H) were generated based on the prediction model.

### SHAP feature analysis

**Fig. 3:**
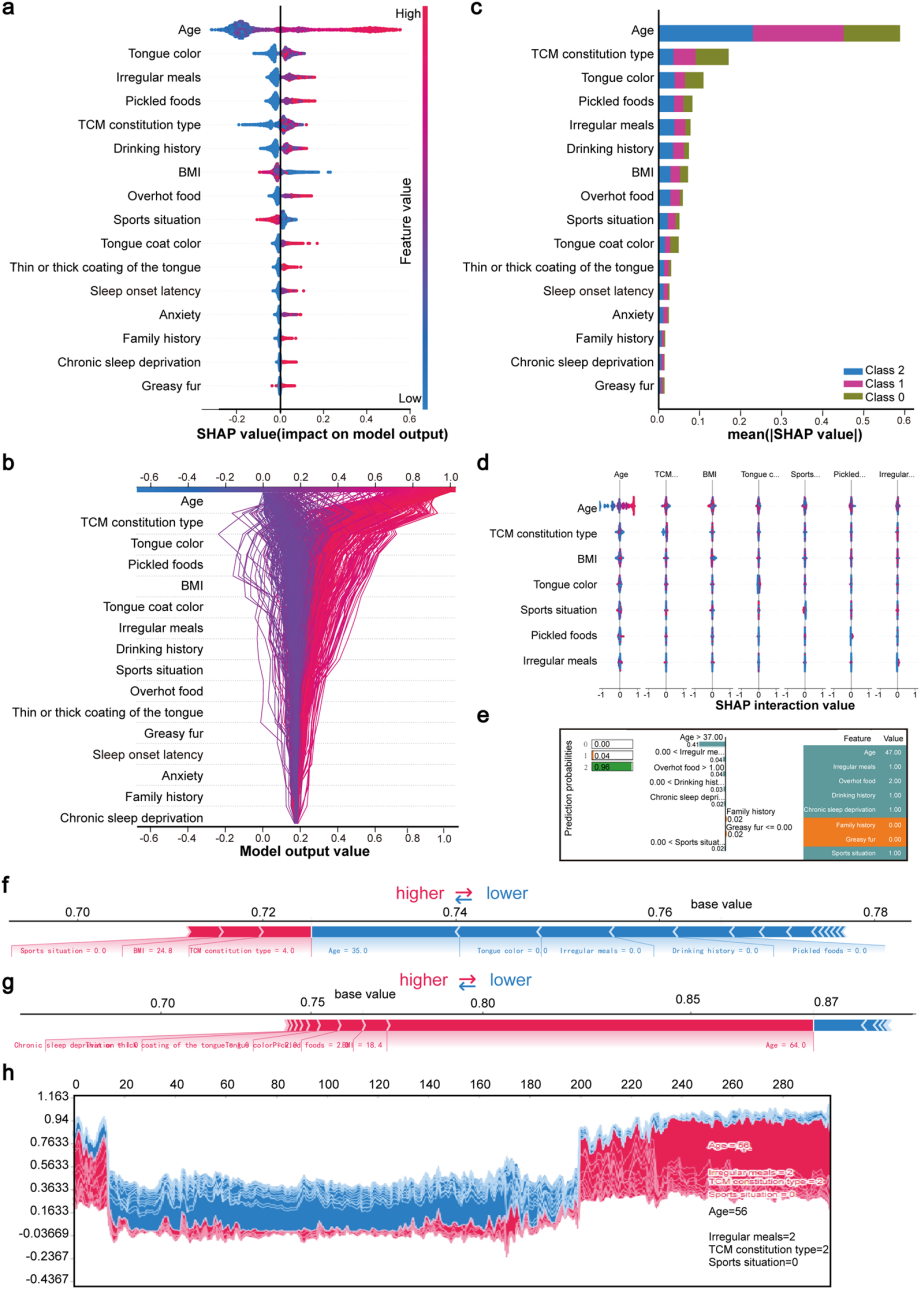
Feature importance analysis diagram of GC risk factors. **a** Importance of features Ranking scatter plots, the impact of each feature on GC can be seen. **b** is a decision diagram that effectively shows how the model arrived at its decisions. **c** Feature importance analysis chart, which shows the proportion of each group. **d** For the interaction diagram of important features, select which features are suitable for combination, and realize fast and accurate pairwise interaction calculation. **e** LIME was used to interpret individual samples. **f** Contribute maps of SHAP features in a non-GC patient. **g** Contribute maps of SHAP characteristics for a GC patient. **h** is the global graph, which is superimposed by the SHAP graph of each sample after rotation of 90 degrees, and the influence of each sample on the output result f(x) of the model can be seen

To further explore the role of the progression of PLGC to GC and enhance the interpretability of the classification model, the SHAP method was employed to analyze the characteristics of the datasets. Results indicated that age had the most significant impact on the model’s predictive outcomes. As age increased, the likelihood of GC prediction for a given sample accordingly rose. A similar trend was observed with an irregular diet: the more irregular the diet, the higher the probability of a GC diagnosis. In contrast, a regular diet reduced the likelihood of GC occurrence (Fig. 3A). The decision plot intuitively displayed the model’s decision-making process (Fig. 3B). This approach was also used for outlier detection. If a line significantly deviated from the others, it may have indicated an outlier. As shown in Fig. 3B, no outliers were detected, confirming the consistency of the developed model.

A feature importance analysis of the dataset representing the progression from PLGC to GC was presented (Fig. 3C). In this figure, Class 0 represented the proportion of healthy individuals, Class 1 referred to PLGC patients, and Class 2 indicated GC patients. Feature interactions were also analyzed, highlighting suitable feature combinations that made rapid and accurate pairwise interaction calculations (Fig. 3D). The prominent red and blue features in the figure effectively enhanced the model's performance by constructing cross features. The primary influence lay along the diagonal, while the interaction effects were located outside the diagonal.

The prediction result for a sample indicated a 96% probability of this patient developing GC (Fig. 3E). The model explained this prediction based on factors such as irregular eating habits, consumption of overhot food, a history of less than 5 years of alcohol consumption, and chronic sleep deprivation. SHAP feature contributions for non-GC patients were also illustrated (Fig. 3F), and it was evident that the reasons for the predicted non-development of GC included a normal tongue color, regular meals, no history of alcohol consumption, and no consumption of pickles. In another sample prediction, factors contributing to the prediction of GC in this patient included frequent consumption of pickles, chronic sleep deprivation, crimson tongue, thick tongue coating,and a BMI of less than 18.5 (Fig. 3G). A global plot, created by rotating each sample's SHAP plot by 90 degrees, was also presented to illustrate the influence of each sample on the model output f(x) (Fig. 3H).

### Establishment of tongue image prediction model based on Swin Transformer

**Fig. 4:**
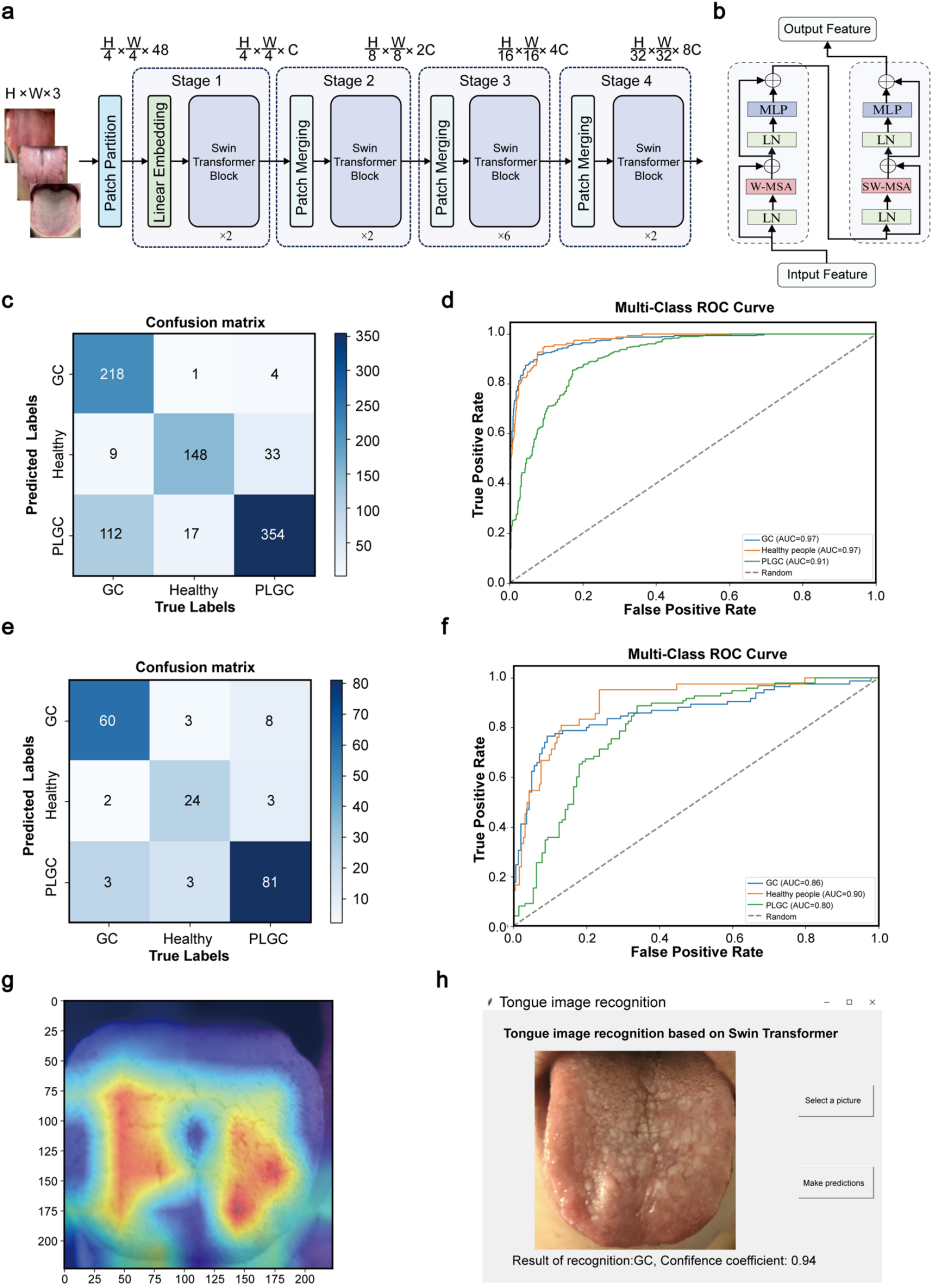
Construction and evaluation of tongue image prediction model based on Swin Transformer**. a** Overall architecture diagram of tongue image model based on Swin Transformer. **b** Swin Transformer Block network structure diagram. **c** Confusion matrix of the train set. **d** ROC curve of the train set. **e** Confusion matrix of the test set. **f** ROC curve of the validation set. **g** is a thermal map, which visually shows how the model evaluates and analyzes the tongue image. **h** Tongue image recognition GUI based on Swin Transformer

In this study, a GC prediction model was constructed based on tongue image analysis. The architecture of the Swin Transformer model employed in this study was shown in Fig. 4A. The model was divided into four stages, each comprising several Swin Transformer modules, where each module was computed using Shift Window-Attention. The detailed network structure of the Swin Transformer Block was shown in Fig. 4B. This model was subsequently applied for predictive analysis. The training set comprised 896 tongue images, with the model correctly predicting 720 images and misclassifying 176, resulting in an accuracy of 80.36% (Fig. 4C). The ROC curve for the training set showed that all AUC values exceeded 0.9, indicating the model’s high predictive accuracy (Fig. 4D).

The confusion matrix of the test set, comprising 225 tongue images, was subsequently analyzed. The model accurately predicted 165 images and misclassified 60, resulting in an accuracy of 73.33% (Fig. 4E). The ROC curve for the test set, with all AUC values exceeding 0.8, indicated that the model maintained a reasonable level of accuracy (Fig. 4F). A heatmap was also generated to visually demonstrate how the model evaluated and analyzed tongue images (Fig. 4G). The important risk factors identified by the model were closely aligned with those recognized by traditional TCM practitioners in clinical settings. This finding demonstrates that the model possesses high interpretability and accuracy, effectively simulating the diagnostic reasoning used by doctors in analyzing and predicting tongue images.

Finally, we presented a GUI developed using the Tkinter module from the Python standard library to facilitate a more intuitive and accessible prediction of GC occurrence based on tongue image analysis (Fig. 4H).

## Discussion

GC in China presents a high incidence and mortality, with over 80% of patients diagnosed at an advanced stage [1]. Early diagnosis of PLGC is crucial for GC prevention and treatment, as it allows intervention before cancer develops. Although PLGC possesses precancerous characteristics, its progression to GC is influenced by multiple factors, including individual lifestyle, environmental factors, and dietary habits. Through in-depth research into the progression of PLGC, we hope to identify the high-risk populations for early intervention to delay or prevent progression to GC. This study examines various factors influencing the progression of PLGC to GC and conducts a comprehensive analysis of the associated risk factors. A predictive model for the progression of PLGC to GC was constructed based on the different scores of various relevant risk factors. The primary objective is to facilitate rapid clinical assessment of risk groups and to develop personalized prevention and treatment strategies for different populations and individuals.

Dynamic nomogram models, widely used as data visualization tools, offer simple, fast, cost-effective, and non-invasive methods for monitoring disease risk and facilitating clinical decision-making. They have been applied in various medical specialties [11, 12]. The dynamic nomogram model was developed in this study, based on logistic regression analysis, achieved an AUC of 0.876 in the validation set for predicting GC risk. The nomogram curve closely matched the ideal curve, indicating strong predictive performance. Utilizing this model to predict GC risk can provide significant clinical benefits for PLGC patients, highlighting the model's clinical value. PLGC patients can monitor their risk in real time based on individual input values, gaining a more intuitive understanding of their risk levels, which can aid in the early prevention of GC.However, the model is limited to binary comparisons, restricting its applicability to datasets requiring multiple categories or more complex relationships. Since the dataset in this study included three categories—healthy people, PLGC, and GC—further analysis using ML was conducted to explore the relationships among these groups.

ML relies on its robust self-learning capabilities to extract deep insights from association rules within clinical data, enabling high-precision, multi-dimensional predictive analyses for personalized assessments of clinical event probabilities in patients [13, 14]. By analyzing the relationship between test indicators and disease, the ML model can provide physicians with a more precise predictive tool, enabling them to implement necessary preventive measures promptly to minimize the risk of GC in patients. This personalized assessment method is of great significance in halting the progression of GC, yielding significant benefits for patients, offering more precise guidance for the prevention and treatment of GC, and ultimately improving the efficiency and quality of clinical practice. In this study, ten ML methods were employed to screen the models with superior accuracy in predicting GC, aiming to enhance the precision and reliability of the prediction results. The training set accuracy of the RF-based prediction model was 99.29%, while the validation set accuracy was 85.65%. Model predictions indicated that Age (> 50 years), low BMI (< 18.5), alcohol drinking history, family history, sleep onset latency (> 30 min), chronic sleep deprivation, anxiety, less exercise, frequent consumption of pickled food, frequent consumption of overhot food, irregular meals, tongue color (pale, crimson, and livid), tongue coating color (yellow, grey, and black), thick fur, greasy fur, and specific TCM constitution types (Yang-deficiency, phlegm-dampness, damp-heat) were important risk factors for GC.

There are various ML algorithmic models that perform well across different prediction tasks. However, the general lack of interpretability in ML algorithms raises concerns about the reliability of their predictions, which significantly limits their application in real-world prediction tasks. SHAP, as one of the interpretability methods for ML, can interpret both individual samples locally and all samples globally, while accounting for feature interactions. This aids in uncovering complex feature relationships, enhancing our understanding of the model’s decision-making process and feature interactions. It is already widely applied in clinical data studies [15, 16]. In this study, the SHAP method was employed to enhance the model’s explainability. It was observed that the primary risk factors for the progression of PLGC to GC included age, TCM constitution type, tongue color, irregular meals, BMI, consumption of pickled foods, and overhot eating habits, which were largely consistent with the predictions made by the RF model. Moreover, SHAP's single-sample interpretation allows for clear visualization of how risk factors are determined, further enhancing the model's interpretability.

These innovative predictive models not only improved early detection rates but also facilitated the personalized screening strategies to enable timely intervention for GC. Additionally, we established a tongue image recognition model based on the Swin Transformer along with a user-friendly GUI, which enabled GC risk recognition by simply selecting any tongue image. Importantly, these models offers the significant advantages such as non-invasiveness, convenience, cost-effectiveness, and strong clinical practicability.

However, there are some limitations to our study. We have not yet conducted a large-scale, long-term follow-up to fully verify the model’s identification power and validity. Such studies require a large number of resources and time, and may face challenges in implementation and operational complexity. Despite these challenges, we hope to make our efforts to enhance the model’s reliability and clinical applicability in the future.

## Conclusion

In summary, we developed a predictive model to estimate the probability of gastric cancer in patients based on various risk factors, which demonstrates high accuracy and reliability. This study analyzed the key risk factors for GC using logistic regression, the construction of a dynamic nomogram prediction model (https://yz6677.shinyapps.io/GC67/), and ML. Age (> 50 years), low BMI (< 18.5), alcohol drinking history, family history, sleep onset latency (> 30 min), chronic sleep deprivation, anxiety, less exercise, frequent consumption of pickled food, frequent consumption of overhot food, irregular meals, tongue color (pale, crimson, and livid), tongue coating color (yellow, grey, and black), thick fur, greasy fur, and specific TCM constitution types (Yang-deficiency, phlegm-dampness, damp-heat), were identified as being associated with an increased risk of PLGC progression to GC. This study found that TCM constitution type and tongue image information can serve as useful indicators for predicting the progression of PLGC. Patients with Yang-deficiency constitution, Phlegm-dampness constitution, and Dampness-heat constitution were found to be more susceptible to GC and should be encouraged to take preventive measures in advance. Since tongue image information serves as an important predictor, this study focused on tongue image prediction for the progression of PLGC to GC and employed the Swin Transformer to build a risk prediction model for tongue image recognition and analysis. This model offers multiple advantages, including non-invasiveness, convenience, and cost-effectiveness. Furthermore, the research team developed a GUI for the PLGC and GC tongue image prediction model, allowing individuals to predict their PLGC and GC risk through this tool. This personalized assessment plays a crucial role in halting the progression of PLGC, benefiting patients by offering more accurate guidance for GC prevention and treatment, ultimately enhancing clinical efficiency and quality of care.

## Supplementary Information

Supplementary material 1.

## Data Availability

The datasets used and analysed during the current study are available from the corresponding author on reasonable request.

## Abbreviations

PLGC: Precancerous lesions of gastric cancer
GC: Gastric cancer
ML: Machine learning
SVC: Support Vector Classification
KNN: K-nearest Neighbor
RF: Random Forest
XGBoost: Extreme Gradient Boosting
GNB: Gaussian Naive Bayes algorithm
MLP: Multilayer Perceptron
GBM: Gradient Boosting Machine
CatBoost: Category Boosting
LightGBM: Light Gradient Boosting Machine
GUI: Graphic User Interface
CNN: Convolutional Neural Networks
NLP: Natural Language Processing
CV: Computer Vision
MLP: Multi-Layer Perceptron
AUC: Area under curve
BMI: Body mass index
ROC: Receiver operating characteristics

## References

[1] Han B, Zheng R, Zeng H, Wang S, Sun K, Chen R, et al. Cancer incidence and mortality in China, 2022. J Natl Cancer Cent. 2024;4(1):47–53. 39036382 10.1016/j.jncc.2024.01.006 PMC11256708

[2] Kinoshita H, Hayakawa Y, Koike K. Metaplasia in the stomach-precursor of gastric cancer? Int J Mol Sci. 2017;18(10):2063. 28953255 10.3390/ijms18102063 PMC5666745

[3] Zhang R, Rabinovitch PS, Mattis AN, Lauwers GY, Choi WT. Gastric intestinal metaplasia in mucosa adjacent to gastric cancers is rarely associated with the aneuploidy that is characteristic of gastric dysplasia or cancer. Am J Surg Pathol. 2021;45(10):1374–81. 34091484 10.1097/PAS.0000000000001764

[4] Chien PL, Liu CF, Huang HT, Jou HJ, Chen SM, Young TG, et al. Application of artificial intelligence in the establishment of an association model between metabolic syndrome, TCM constitution, and the guidance of medicated diet care. Evid Based Complement Alternat Med. 2021;2021:5530717. 34007288 10.1155/2021/5530717 PMC8110390

[5] Liu Q, Li Y, Yang P, Liu Q, Wang C, Chen K, et al. A survey of artificial intelligence in tongue image for disease diagnosis and syndrome differentiation. Digit Health. 2023;9:20552076231191044. 37559828 10.1177/20552076231191044 PMC10408356

[6] Rigatti SJ. Random Forest. J Insur Med. 2017;47(1):31–9. 28836909 10.17849/insm-47-01-31-39.1

[7] Kou FR, Zhang YZ, Xu WR. Prognostic nomograms for predicting overall survival and cause-specific survival of signet ring cell carcinoma in colorectal cancer patients. World J Clin Cases. 2021;9(11):2503–18. 33889615 10.12998/wjcc.v9.i11.2503 PMC8040180

[8] Peeperkorn S, Meulemans J, Van Lierde C, Laenen A, Valstar MH, Balm AJM, et al. Validated prognostic nomograms for patients with parotid carcinoma predicting 2- and 5-year tumor recurrence-free interval probability. Front Oncol. 2020;10:1535. 32984008 10.3389/fonc.2020.01535 PMC7477337

[9] Liu Z, Lin Y, Cao Y, Hu H, Wei Y, Zhang Z, et al. Swin Transformer: Hierarchical Vision Transformer using Shifted Windows. 2021:arXiv:2103.14030.

[10] Bajic F, Orel O, Habijan M. A multi-purpose shallow convolutional neural network for chart images. Sensors. 2022;22(20):7695. 36298046 10.3390/s22207695 PMC9612160

[11] Chen D, Zhao J, Ma M, Jiang L, Tan Y, Wan X. Dynamic nomogram for predicting acute kidney injury in patients with community-acquired pneumonia. BMJ Open Respir Res. 2023;10(1): e001495. 37739457 10.1136/bmjresp-2022-001495 PMC10533799

[12] Zhuo X, Yu J, Chen Z, Lin Z, Huang X, Chen Q, et al. Dynamic nomogram for predicting lateral cervical lymph node metastasis in papillary thyroid carcinoma. Otolaryngol Head Neck Surg. 2022;166(3):444–53. 34058905 10.1177/01945998211009858

[13] Handelman GS, Kok HK, Chandra RV, Razavi AH, Lee MJ, Asadi H. eDoctor: machine learning and the future of medicine. J Intern Med. 2018;284(6):603–19. 30102808 10.1111/joim.12822

[14] Sultan AS, Elgharib MA, Tavares T, Jessri M, Basile JR. The use of artificial intelligence, machine learning and deep learning in oncologic histopathology. J Oral Pathol Med. 2020;49(9):849–56. 32449232 10.1111/jop.13042

[15] Debjit K, Islam MS, Rahman MA, Pinki FT, Nath RD, Al-Ahmadi S, et al. An improved machine-learning approach for COVID-19 prediction using harris hawks optimization and feature analysis using SHAP. Diagnostics. 2022;12(5):1023. 35626179 10.3390/diagnostics12051023 PMC9139459

[16] Park J, Kim J, Ryu D, Choi HY. Factors related to steroid treatment responsiveness in thyroid eye disease patients and application of SHAP for feature analysis with XGBoost. Front Endocrinol. 2023;14:1079628. 10.3389/fendo.2023.1079628 PMC9928572 36817584

