# Supplementary material for: Construction of machine learning-based models for screening the high-risk patients with gastric precancerous lesions
Source: Chin Med. 2025 Jan 7;20:7. doi: 10.1186/s13020-025-01059-4 (PMC11705657; doi:10.1186/s13020-025-01059-4)
Supplement: Supplementary file 1 — Supplementary material 1. [file 13020_2025_1059_MOESM1_ESM.docx]

**Construction of machine learning-based models for screening the high-risk patients with gastric precancerous lesions**

Shuxian Yu^1,3, #^, Haiyang Jiang^1, #^, Jing Xia^1^, Jie Gu^1^, Mengting Chen^1^, Yan Wang^1^, Xiaohong Zhao^1^, Zehua Liao^1^, Puhua Zeng^4, *^, Tian Xie^1, 2, *^, Xinbing Sui^1, 2,^ ^*^

^1^School of Pharmacy, Hangzhou Normal University, Hangzhou, China

^2^Department of Medical Oncology, The Affiliated Hospital of Hangzhou Normal University, Hangzhou, China

^3^The First Affiliated Hospital of Zhejiang Chinese Medicine University, Hangzhou, China

^4^The Affiliated Hospital of Hunan Academy of Traditional Chinese Medicine, Changsha, Hunan, China

*Correspondence: Xinbing Sui, Email: [hzzju@hznu.edu.cn](mailto:hzzju@hznu.edu.cn) or Tian Xie, Email: [xbs@hznu.edu.cn](mailto:xbs@hznu.edu.cn) or Puhua Zeng, Email: zph120@126.com

^#^These authors contribute equally.

**Figures:**

**Supplementary Figure 1 | Flowchart of Participant Enrollment.**

**
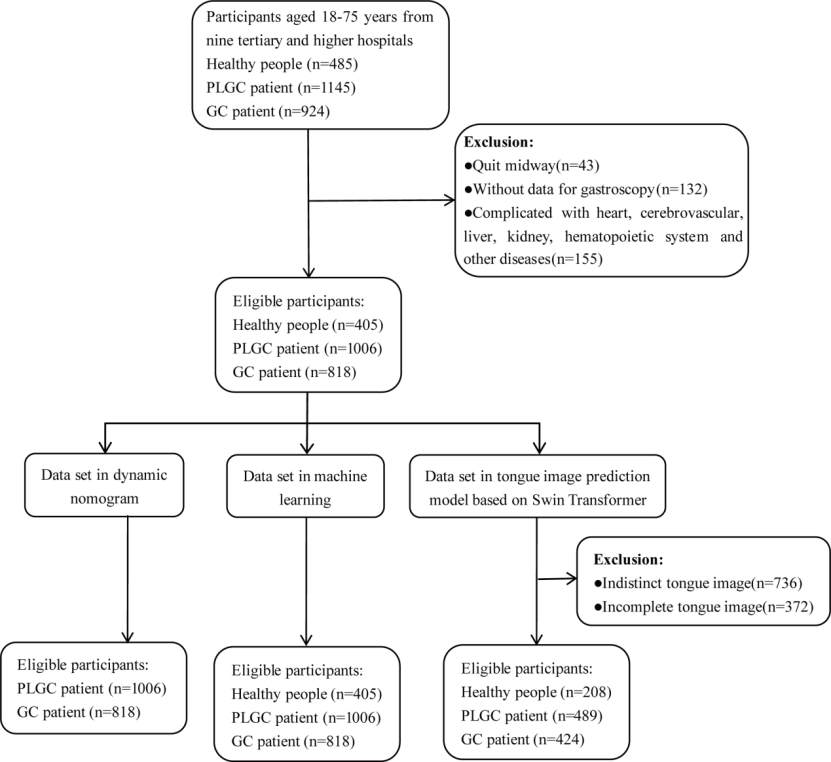
**

**Tables:**

**Supplementary Table 1 | Univariate analysis of basic conditions of healthy population and PLGC patients [n (%)].**

| **Factor** | **Category** | **Healthy people (n=405)** | **PLGC**  **(n=1006)** | **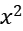** | ***P*** |
| --- | --- | --- | --- | --- | --- |
| Gender  Age (years)  BMI | Male  Female  ≤30  31 ~ 40  41 ~ 50  >50  <18.5  18.5 ~ 24.0  >24.0 | 204 (50.3)  201 (49.6)  304 (75.0)  72 (17.7)  16 (3.9)  13 (3.2)  18 (4.4)  371 (91.6)  16 (3.9) | 518 (51.4)  488 (48.5)  111 (11.0)  487 (48.4)  276 (27.4)  132 (13.1)  189 (18.7)  721 (71.6)  96 (9.5) | 0.145  575.4  66.69 | 0.703  0.000  0.000 |

**Supplementary Table 2 | Univariate analysis of basic conditions of healthy population and GC patients [n (%)].**

| **Factor** | **Category** | **Healthy people (n=405)** | **GC**  **(n=818)** | **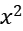** | ***P*** |
| --- | --- | --- | --- | --- | --- |
| Gender  Age (years)  BMI | Male  Female  ≤30  31~40  41~50  >50  <18.5  18.5 ~ 24.0  >24.0 | 204 (50.3)  201 (49.6)  304 (75.0)  72 (17.7)  16 (3.9)  13 (3.2)  18 (4.4)  371 (91.6)  16 (3.9) | 458 (55.9)  360 (44.0)  12 (1.4)  78 (9.5)  154 (18.8)  574 (70.1)  272 (33.2)  484 (59.1)  62 (7.5) | 3.446  879.0  141.2 | 0.063  0.000  0.000 |

**Supplementary Table 3 | Univariate analysis of PLGC and basic information of GC patients [n (%)].**

| **Factor** | **Category** | **PLGC**  **(n=1006)** | **GC**  **(n=818)** | **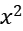** | ***P*** |
| --- | --- | --- | --- | --- | --- |
| Gender  Age (years)  BMI | Male  Female  ≤30  31 ~ 40  41 ~ 50  >50  <18.5  18.5 ~ 24.0  >24.0 | 518 (51.4)  488 (48.5)  111 (11.0)  487 (48.4)  276 (27.4)  132 (13.1)  189 (18.7)  721 (71.6)  96 (9.5) | 458 (55.9)  360 (44.0)  12 (1.4)  78 (9.5)  154 (18.8)  574 (70.1)  272 (33.2)  484 (59.1)  62 (7.5) | 3.671  674.9  50.03 | 0.055  0.000  0.000 |

**Supplementary Table 4 | Univariate analysis of smoking and drinking among healthy people and PLGC patients [n (%)].**

| **Factor** | **Category** | **Healthy people (n=405)** | **PLGC**  **(n=1006)** | **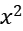** | ***P*** |
| --- | --- | --- | --- | --- | --- |
| Alcohol drinking history  Smoking history | No  <5 years  5 ~ 10 years  >10 years  No  <5 years  5 ~ 10 years  >10 years | 269 (66.4)  67 (16.5)  49 (12.0)  20 (4.9)  291 (71.8)  39 (9.6)  45 (11.1)  30 (7.4) | 567 (56.3)  169 (16.7)  172 (17.0)  98 (9.7)  699 (69.4)  112 (11.1)  110 (10.9)  85 (8.4) | 17.51  1.234 | 0.000  0.745 |

**Supplementary Table 5 | Univariate analysis of smoking and drinking among healthy people and GC patients [n (%)].**

| **Factor** | **Category** | **Healthy people(n=405)** | **GC**  **(n=818)** | **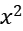** | ***P*** |
| --- | --- | --- | --- | --- | --- |
| Alcohol drinking history  Smoking history | No  <5 years  5 ~ 10 years  >10 years  No  <5 years  5 ~ 10 years  >10 years | 269 (66.4)  67 (16.5)  49 (12.0)  20 (4.9)  291 (71.8)  39 (9.6)  45 (11.1)  30 (7.4) | 335 (40.9)  187 (22.8)  177 (21.6)  119 (14.5)  554 (67.7)  81 (9.9)  85 (10.3)  98 (11.9) | 76.12  6.233 | 0.000  0.101 |

**Supplementary Table 6 | Univariate analysis of smoking and alcohol drinking history in PLGC and GC patients [n (%)].**

| **Factor** | **Category** | **PLGC**  **(n=1006)** | **GC**  **(n=818)** | **χ^2^** | ***P*** |
| --- | --- | --- | --- | --- | --- |
| Alcohol drinking history  Smoking history | No  <5 years  5 ~ 10 years  >10 years  No  <5 years  5 ~ 10 years  >10 years | 567 (56.3)  169 (16.7)  172 (17.0)  98 (9.7)  699 (69.4)  112 (11.1)  110 (10.9)  85 (8.4) | 335 (40.9)  187 (22.8)  177 (21.6)  119 (14.5)  554 (67.7)  81 (9.9)  85 (10.3)  98 (11.9) | 43.77  6.580 | 0.000  0.086 |

**Supplementary Table 7 | Univariate analysis of disease history between healthy population and PLGC patients [n (%)].**

| **Factor** | **Category** | **Healthy people (n=405)** | **PLGC**  **(n=1006)** | **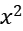** | ***P*** |
| --- | --- | --- | --- | --- | --- |
| Hypertension  Family history  Diabetes  Anaemia | no  yes  no  yes  no  yes  no  yes | 346 (85.4)  59 (14.5)  346 (85.4)  59 (14.5)  376 (91.8)  29 (7.1)  358 (88.3)  47 (11.6) | 829 (82.4)  177 (17.5)  779 (77.4)  227 (22.5)  926 (92.0)  80 (7.9)  854 (84.8)  152 (15.1) | 1.899  11.43  0.254  2.927 | 0.168  0.000  0.614  0.087 |

**Supplementary Table 8 | Univariate analysis of disease history between healthy population and GC patients [n (%)].**

| **Factor** | **Category** | **Healthy people (n=405)** | **GC**  **(n=818)** | **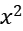** | ***P*** |
| --- | --- | --- | --- | --- | --- |
| Hypertension  Family history  Diabetes  Anaemia | no  yes  no  yes  no  yes  no  yes | 346 (85.4)  59 (14.5)  346 (85.4)  59 (14.5)  376 (91.8)  29 (7.1)  358 (88.3)  47 (11.6) | 671 (82.0)  147 (17.9)  562 (68.7)  256 (31.2)  736 (89.9)  82 (10.0)  673 (82.2)  145 (17.7) | 2.239  39.64  2.692  7.669 | 0.135  0.000  0.101  0.006 |

**Supplementary Table 9 | Univariate analysis of disease history in patients with PLGC and GC [n (%)].**

| **Factor** | **Category** | **PLGC**  **(n=1006)** | **GC**  **(n=818)** | **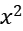** | ***P*** |
| --- | --- | --- | --- | --- | --- |
| Hypertension  Family history  Diabetes  Anaemia | no  yes  no  yes  no  yes  no  yes | 829 (82.4)  177 (17.5)  779 (77.4)  227 (22.5)  926 (92.0)  80 (7.9)  854 (84.8)  152 (15.1) | 671 (82.0)  147 (17.9)  562 (68.7)  256 (31.2)  736 (89.9)  82 (10.0)  673 (82.2)  145 (17.7) | 0.044  17.67  2.394  2.266 | 0.834  0.000  0.122  0.132 |

**Supplementary Table 10 | Univariate analysis of sleep status between healthy people and PLGC patients [n (%)].**

| **Factor** | **Category** | **Healthy people (n=405)** | **PLGC**  **(n=1006)** | **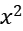** | ***P*** |
| --- | --- | --- | --- | --- | --- |
| Sleep onset latency  Insomnia  Irregular sleep  Stay up late  Somnolence  Chronic sleep deprivation | <30min  30 ~ 60min  >60min  No  <3 months  ≥3 months  No  Yes  <11 p.m.  11 p.m. ~ 12 a.m.  12 a.m. ~ 1.a.m.  >1 a.m.  No  Yes  No  Yes | 302 (74.5)  64 (15.8)  39 (9.6)  327 (80.7)  52 (12.8)  26 (6.4)  355 (87.6)  50 (12.3)  243 (60.0)  91 (22.4)  44 (10.8)  27 (6.6)  342 (84.4)  63 (15.5)  365 (90.1)  40 (9.8) | 725 (72.0)  192 (19.0)  89 (8.8)  799 (79.4)  153 (15.2)  54 (5.3)  821 (81.6)  185 (18.3)  553 (54.9)  257 (25.5)  122 (12.1)  74 (7.3)  883 (87.7)  123 (12.2)  871 (86.5)  135 (13.4) | 2.158  1.742  7.599  2.987  2.796  3.336 | 0.339  0.419  0.006  0.394  0.095  0.068 |

**Supplementary Table 11 | Univariate analysis of sleep status between healthy people and GC patients [n (%)].**

| **Factor** | **Category** | **Healthy people (n=405)** | **GC**  **(n=818)** | **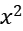** | ***P*** |
| --- | --- | --- | --- | --- | --- |
| Sleep onset latency  Insomnia  Irregular sleep  Stay up late  Somnolence  Chronic sleep deprivation | <30min  30 ~ 60min  >60min  No  <3 months  ≥3 months  No  Yes  <11 p.m.  11 p.m. ~ 12 a.m.  12 a.m. ~ 1.a.m.  >1 a.m.  No  Yes  No  Yes | 302 (74.5)  64 (15.8)  39 (9.6)  327 (80.7)  52 (12.8)  26 (6.4)  355 (87.6)  50 (12.3)  243 (60.0)  91 (22.4)  44 (10.8)  27 (6.6)  342 (84.4)  63 (15.5)  365 (90.1)  40 (9.8) | 520 (63.5)  201 (24.5)  97 (11.8)  664 (81.1)  102 (12.4)  52 (6.3)  657 (80.3)  161 (19.6)  490 (59.9)  197 (24.0)  86 (10.5)  45 (5.5)  699 (85.4)  119 (14.5)  660 (80.6)  158 (19.3) | 15.70  0.037  10.21  0.956  0.217  17.79 | 0.000  0.982  0.001  0.812  0.641  0.000 |

**Supplementary Table 12 | Univariate analysis of sleep status in PLGC and GC patients [n (%)].**

| **Factor** | **Category** | **PLGC**  **(n=1006)** | **GC**  **(n=818)** | **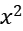** | ***P*** |
| --- | --- | --- | --- | --- | --- |
| Sleep onset latency  Insomnia  Irregular sleep  Stay up late  Somnolence  Chronic sleep deprivation | <30min  30 ~ 60min  >60min  No  <3 months  ≥3 months  No  Yes  <11 p.m.  11 p.m. ~ 12 a.m.  12 a.m. ~ 1.a.m.  >1 a.m.  No  Yes  No  Yes | 725 (72.0)  192 (19.0)  89 (8.8)  799 (79.4)  153 (15.2)  54 (5.3)  821 (81.6)  185 (18.3)  553 (54.9)  257 (25.5)  122 (12.1)  74 (7.3)  883 (87.7)  123 (12.2)  871 (86.5)  135 (13.4) | 520 (63.5)  201 (24.5)  97 (11.8)  664 (81.1)  102 (12.4)  52 (6.3)  657 (80.3)  161 (19.6)  490 (59.9)  197 (24.0)  86 (10.5)  45 (5.5)  699 (85.4)  119 (14.5)  660 (80.6)  158 (19.3) | 15.09  3.353  0.490  5.716  2.112  11.63 | 0.000  0.187  0.484  0.126  0.146  0.000 |

**Supplementary Table 13 | Univariate analysis of psychosocial factors between healthy population and PLGC patients [n (%)].**

| **Factor** | **Category** | **Healthy people (n=405)** | **PLGC**  **(n=1006)** | **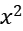** | ***P*** |
| --- | --- | --- | --- | --- | --- |
| Tension  Depression  Anxiety  High work pressure | Never  Minor  Severe  Never  Minor  Severe  Never  Minor  Severe  Never  Minor  Severe | 326 (80.4)  56 (13.8)  23 (5.6)  284 (70.1)  87 (21.4)  34 (8.3)  345 (85.1)  44 (10.8)  16 (3.9)  199 (49.1)  120 (21.6)  86 (21.2) | 811 (80.6)  142 (14.1)  53 (5.2)  674 (66.9)  245 (24.3)  87 (8.6)  809 (80.4)  130 (12.9)  67 (6.6)  463 (46.0)  319 (31.7)  224 (22.2) | 0.108  1.450  5.398  1.138 | 0.947  0.484  0.067  0.556 |

**Supplementary Table 14 | Univariate analysis of psychosocial factors between healthy population and GC patients [n (%)].**

| **Factor** | **Category** | **Healthy people (n=405)** | **GC**  **(n=818)** | **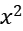** | ***P*** |
| --- | --- | --- | --- | --- | --- |
| Tension  Depression  Anxiety  High work pressure | Never  Minor  Severe  Never  Minor  Severe  Never  Minor  Severe  Never  Minor  Severe | 326 (80.4)  56 (13.8)  23 (5.6)  284 (70.1)  87 (21.4)  34 (8.3)  345 (85.1)  44 (10.8)  16 (3.9)  199 (49.1)  120 (21.6)  86 (21.2) | 655 (80.0)  107 (13.0)  56 (6.8)  525 (64.1)  205 (25.0)  88 (10.7)  598 (73.1)  150 (18.3)  70 (8.5)  399 (48.7)  240 (29.3)  179 (21.8) | 0.690  4.416  22.84  0.067 | 0.708  0.109  0.000  0.967 |

**Supplementary Table 15 | Univariate analysis of psychosocial factors between PLGC patients and GC patients [n (%)].**

| **Factor** | **Category** | **PLGC**  **(n=1006)** | **GC**  **(n=818)** | **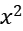** | ***P*** |
| --- | --- | --- | --- | --- | --- |
| Tension  Depression  Anxiety  High work pressure | Never  Minor  Severe  Never  Minor  Severe  Never  Minor  Severe  Never  Minor  Severe | 811 (80.6)  142 (14.1)  53 (5.2)  674 (66.9)  245 (24.3)  87 (8.6)  809 (80.4)  130 (12.9)  67 (6.6)  463 (46.0)  319 (31.7)  224 (22.2) | 655 (80.0)  107 (13.0)  56 (6.8)  525 (64.1)  205 (25.0)  88 (10.7)  598 (73.1)  150 (18.3)  70 (8.5)  399 (48.7)  240 (29.3)  179 (21.8) | 2.249  2.729  13.91  1.581 | 0.325  0.255  0.001  0.454 |

**Supplementary Table 16 | Univariate analysis of sports situation between healthy people and PLGC patients [n (%)].**

| **Factor** | **Category** | **Healthy people (n=405)** | **PLGC**  **(n=1006)** | **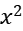** | ***P*** |
| --- | --- | --- | --- | --- | --- |
| Sports situation | Less  Everyday  Every 3-5 days  Once a week | 126 (31.1)  79 (19.5)  79 (19.5)  121 (29.8) | 414 (41.1)  175 (17.3)  181 (17.9)  236 (23.4) | 13.38 | 0.004 |

**Supplementary Table 17 | Univariate analysis of sports situation between healthy people and GC patients [n (%)].**

| **Factor** | **Category** | **Healthy people**  **(n=405)** | **GC**  **(n=818)** | **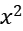** | ***P*** |
| --- | --- | --- | --- | --- | --- |
| Sports situation | Less  Everyday  Every 3-5 days  Once a week | 126 (31.1)  79 (19.5)  79 (19.5)  121 (29.8) | 385 (47.0)  217 (26.5)  109 (13.3)  107 (13.0) | 69.74 | 0.000 |

**Supplementary Table 18 | Univariate analysis of sports situation in PLGC and GC patients [n (%)].**

| **Factor** | **Category** | **PLGC**  **(n=1006)** | **GC**  **(n=818)** | **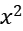** | ***P*** |
| --- | --- | --- | --- | --- | --- |
| Sports situation | Less  Everyday  Every 3-5 days  Once a week | 414 (41.1)  175 (17.3)  181 (17.9)  236 (23.4) | 385 (47.0)  217 (26.5)  109 (13.3)  107 (13.0) | 53.13 | 0.000 |

**Supplementary Table 19 | Univariate analysis of dietary status between healthy people and PLGC patients [n (%)].**

| **Factor** | **Category** | **Healthy people (n=405)** | **PLGC**  **(n=1006)** | **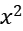** | ***P*** |
| --- | --- | --- | --- | --- | --- |
| Cold food  Pickled food  Spicy food  Fast eating  Greasy food  High-salt diet  Overhot food  Irregular meals  Smoked food | Never  Sometimes  Often  Never  Sometimes  Often  Never  Sometimes  Often  Never  Sometimes  Often  Never  Sometimes  Often  No  Yes  Never  Sometimes  Often  Never  Sometimes  Often  Never  Sometimes  Often | 319 (78.7)  58 (14.3)  28 (6.9)  347 (85.6)  39 (9.6)  19 (4.6)  276 (68.1)  87 (21.4)  42 (10.3)  269 (66.4)  91 (22.4)  45 (11.1)  306 (75.5)  69 (17.0)  30 (7.4)  342 (84.4)  63 (15.5)  335 (82.7)  45 (11.1)  25 (6.1)  331 (81.7)  55 (13.5)  19 (4.6)  340 (83.9)  39 (9.6)  26 (6.4) | 741 (73.6)  167 (16.6)  98 (9.7)  676 (67.1)  209 (20.7)  121 (12.0)  679 (67.4)  206 (20.7)  118 (11.7)  677 (67.2)  209 (20.7)  120 (11.9)  757 (75.2)  180 (17.8)  69 (6.8)  854 (84.8)  152 (15.1)  770 (76.5)  149 (14.8)  87 (8.6)  727 (72.2)  189 (18.7)  90 (8.9)  849 (84.3)  106 (10.5)  51 (5.0) | 4.529  49.68  0.626  0.588  0.247  0.045  6.510  14.74  1.203 | 0.104  0.000  0.731  0.745  0.884  0.833  0.039  0.000  0.548 |

**Supplementary Table 20 | Univariate analysis of dietary status between healthy people and GC patients [n (%)].**

| **Factor** | **Category** | **Healthy people (n=405)** | **GC**  **(n=818)** | **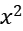** | ***P*** |
| --- | --- | --- | --- | --- | --- |
| Cold food  Pickled food  Spicy food  Fast eating  Greasy food  High-salt diet  Overhot food  Irregular meals  Smoked food | Never  Sometimes  Often  Never  Sometimes  Often  Never  Sometimes  Often  Never  Sometimes  Often  Never  Sometimes  Often  No  Yes  Never  Sometimes  Often  Never  Sometimes  Often  Never  Sometimes  Often | 319 (78.7)  58 (14.3)  28 (6.9)  347 (85.6)  39 (9.6)  19 (4.6)  276 (68.1)  87 (21.4)  42 (10.3)  269 (66.4)  91 (22.4)  45 (11.1)  306 (75.5)  69 (17.0)  30 (7.4)  342 (84.4)  63 (15.5)  335 (82.7)  45 (11.1)  25 (6.1)  331 (81.7)  55 (13.5)  19 (4.6)  340 (83.9)  39 (9.6)  26 (6.4) | 624 (76.2)  115 (14.0)  79 (9.6)  420 (51.3)  234 (28.6)  164 (20.0)  583 (71.2)  150 (18.3)  85 (10.4)  511 (62.4)  184 (22.4)  123 (15.0)  651 (79.5)  117 (14.3)  50 (6.1)  702 (85.8)  116 (14.1)  491 (60.0)  174 (21.2)  153 (18.7)  426 (52.0)  270 (33.0)  122 (14.9)  702 (85.8)  78 (9.5)  38 (4.6) | 2.561  137.3  1.758  3.702  2.588  0.410  65.49  101.5  1.743 | 0.278  0.000  0.415  0.157  0.274  0.522  0.000  0.000  0.418 |

**Supplementary Table 21 | Univariate analysis of dietary status between PLGC and GC patients [n (%)].**

| **Factor** | **Category** | **PLGC**  **(n=1006)** | **GC**  **(n=818)** | **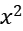** | ***P*** |
| --- | --- | --- | --- | --- | --- |
| Cold food  Pickled food  Spicy food  Fast eating  Greasy food  High-salt diet  Overhot food  Irregular meals  Smoked food | Never  Sometimes  Often  Never  Sometimes  Often  Never  Sometimes  Often  Never  Sometimes  Often  Never  Sometimes  Often  No  Yes  Never  Sometimes  Often  Never  Sometimes  Often  Never  Sometimes  Often | 741 (73.6)  167 (16.6)  98 (9.7)  676 (67.1)  209 (20.7)  121 (12.0)  679 (67.4)  206 (20.7)  118 (11.7)  677 (67.2)  209 (20.7)  120 (11.9)  757 (75.2)  180 (17.8)  69 (6.8)  854 (84.8)  152 (15.1)  770 (76.5)  149 (14.8)  87 (8.6)  727 (72.2)  189 (18.7)  90 (8.9)  849 (84.3)  106 (10.5)  51 (5.0) | 624 (76.2)  115 (14.0)  79 (9.6)  420 (51.3)  234 (28.6)  164 (20.0)  583 (71.2)  150 (18.3)  85 (10.4)  511 (62.4)  184 (22.4)  123 (15.0)  651 (79.5)  117 (14.3)  50 (6.1)  702 (85.8)  116 (14.1)  491 (60.0)  174 (21.2)  153 (18.7)  426 (52.0)  270 (33.0)  122 (14.9)  702 (85.8)  78 (9.5)  38 (4.6) | 2.304  48.84  2.710  5.504  5.054  0.310  63.11  79.17  0.723 | 0.316  0.000  0.258  0.064  0.079  0.578  0.000  0.000  0.697 |

**Supplementary Table 22 | Univariate analysis of tongue image information between healthy population and PLGC patients [n (%)].**

| **Factor** | **Category** | **Healthy people (n=405)** | **PLGC**  **(n=1006)** | **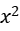** | ***P*** |
| --- | --- | --- | --- | --- | --- |
| Tongue color  Tongue coating color  Thin or thick coating  Greasy fur  Curdy fur  Peeling tongue fur  Plumpness and slenderness of tongue  Tongue local features  Abnormal tongue situation | Light red tongue  Pale tongue  Crimson tongue  Livid tongue  White tongue fur  Yellow tongue fur  Grey tongue fur  Black tongue fur  Thin fur  Thick fur  No  Yes  No  Yes  No  Yes  Slender tongue  Bulgy tongue  Normal  Teeth-printed tongue  Spotted tongue  Cracked tongue  Normal  The edge of the tongue is red  Petechial ecchymosis | 317 (78.2)  70(17.2)  18 (4.4)  0 (0)  340 (83.9)  65 (16.0)  0 (0)  0 (0)  345 (85.1)  60 (14.8)  361 (89.1)  44 (10.8)  368 (90.8)  37 (9.1)  398 (98.2)  7 (1.7)  329 (81.2)  76 (18.7)  311 (76.7)  33 (8.1)  42 (10.3)  19 (4.6)  345 (85.1)  51 (12.5)  9 (2.2) | 477 (47.4)  307 (30.5)  149 (14.8)  73 (7.2)  666 (66.2)  266 (26.4)  66 (6.5)  8 (0.7)  714 (70.9)  292 (29.0)  828 (82.3)  178 (17.6)  867 (86.1)  139 (13.8)  987 (98.1)  19 (1.8)  787 (78.2)  219 (21.7)  779 (77.4)  86 (8.5)  113 (11.2)  28 (2.7)  831 (82.6)  147 (14.6)  28 (2.7) | 123.4  55.84  31.15  10.16  5.796  0.041  1.576  3.422  1.417 | 0.000  0.000  0.000  0.001  0.016  0.839  0.209  0.331  0.492 |

**Supplementary Table 23 | Univariate analysis of tongue image information between healthy population and GC patients [n (%)].**

| **Factor** | **Category** | **Healthy people (n=405)** | **GC**  **(n=818)** | **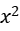** | ***P*** |
| --- | --- | --- | --- | --- | --- |
| Tongue color  Tongue coating color  Thin or thick coating  Greasy fur  Curdy fur  Peeling tongue fur  Plumpness and slenderness of tongue  Tongue local features  Abnormal tongue situation | Light red tongue  Pale tongue  Crimson tongue  Livid tongue  White tongue fur  Yellow tongue fur  Grey tongue fur  Black tongue fur  Thin fur  Thick fur  No  Yes  No  Yes  No  Yes  Slender tongue  Bulgy tongue  Normal  Teeth-printed tongue  Spotted tongue  Cracked tongue  Normal  The edge of the tongue is red  Petechial ecchymosis | 317 (78.2)  70 (17.2)  18 (4.4)  0 (0)  340 (83.9)  65 (16.0)  0 (0)  0 (0)  345 (85.1)  60 (14.8)  361 (89.1)  44 (10.8)  368 (90.8)  37 (9.1)  398 (98.2)  7 (1.7)  329 (81.2)  76 (18.7)  311 (76.7)  33 (8.1)  42 (10.3)  19 (4.6)  345 (85.1)  51 (12.5)  9 (2.2) | 262 (32.0)  309 (37.7)  186 (22.7)  61 (7.4)  431 (52.6)  291 (35.5)  78 (9.5)  18 (2.2)  501 (61.2)  317 (38.7)  594 (72.6)  224 (27.3)  718 (87.7)  100 (12.2)  800 (97.7)  18 (2.2)  627 (76.6)  191 (23.3)  593 (72.4)  84 (10.2)  109 (13.3)  32 (3.9)  652 (79.7)  142 (17.3)  24 (2.9) | 243.6  125.0  72.80  43.20  2.599  0.302  3.336  4.260  5.406 | 0.000  0.000  0.000  0.000  0.107  0.583  0.068  0.235  0.067 |

**Supplementary Table 24 | Univariate analysis of tongue image information between PLGC patients and GC patients [n (%)].**

| **Factor** | **Category** | **PLGC**  **(n=1006)** | **GC**  **(n=818)** | **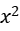** | ***P*** |
| --- | --- | --- | --- | --- | --- |
| Tongue color  Tongue coating color  Thin or thick coating  Greasy fur  Curdy fur  Peeling tongue fur  Plumpness and slenderness of tongue  Tongue local features  Abnormal tongue situation | Light red tongue  Pale tongue  Crimson tongue  Livid tongue  White tongue fur  Yellow tongue fur  Grey tongue fur  Black tongue fur  Thin fur  Thick fur  No  Yes  No  Yes  No  Yes  Slender tongue  Bulgy tongue  Normal  Teeth-printed tongue  Spotted tongue  Cracked tongue  Normal  The edge of the tongue is red  Petechial ecchymosis | 477 (47.4)  307 (30.5)  149 (14.8)  73 (7.2)  666 (66.2)  266 (26.4)  66 (6.5)  8 (0.7)  714 (70.9)  292 (29.0)  828 (82.3)  178 (17.6)  867 (86.1)  139 (13.8)  987 (98.1)  19 (1.8)  787 (78.2)  219 (21.7)  779 (77.4)  86 (8.5)  113 (11.2)  28 (2.7)  831 (82.6)  147 (14.6)  28 (2.7) | 262 (32.0)  309 (37.7)  186 (22.7)  61 (7.4)  431 (52.6)  291 (35.5)  78 (9.5)  18 (2.2)  501 (61.2)  317 (38.7)  594 (72.6)  224 (27.3)  718 (87.7)  100 (12.2)  800 (97.7)  18 (2.2)  627 (76.6)  191 (23.3)  593 (72.4)  84 (10.2)  109 (13.3)  32 (3.9)  652 (79.7)  142 (17.3)  24 (2.9) | 48.86  37.33  19.19  24.65  1.004  0.221  0.647  6.267  2.651 | 0.000  0.000  0.000  0.000  0.316  0.639  0.421  0.099  0.266 |

**Supplementary Table 25 | Univariate analysis of TCM constitution type between healthy people and PLGC patients [n (%)].**

| **Factor** | **Category** | **Healthy people (n=405)** | **PLGC**  **(n=1006)** | **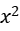** | ***P*** |
| --- | --- | --- | --- | --- | --- |
| TCM constitution type | Balanced constitution  Qi-deficiency constitution  Yang-deficiency constitution  Yin-deficiency constitution  Phlegm-dampness constitution  Dampness-heat constitution  Blood stasis constitution  Qi stagnation constitution  Inherited special constitution | 263 (64.9)  38 (9.3)  23 (5.6)  28 (6.9)  15 (3.7)  15 (3.7)  0 (0)  21 (5.1)  2 (0.4) | 146 (14.5)  257 (25.5)  129 (12.8)  78 (7.7)  115 (11.4)  87 (8.6)  39 (3.8)  144 (14.3)  11 (1.0) | 369.2 | 0.000 |

**Supplementary Table 26 | Univariate analysis of TCM constitution type between healthy people and GC patients [n (%)].**

| **Factor** | **Category** | **Healthy people (n=405)** | **GC**  **(n=818)** | **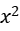** | ***P*** |
| --- | --- | --- | --- | --- | --- |
| TCM constitution type | Balanced constitution  Qi-deficiency constitution  Yang-deficiency constitution  Yin-deficiency constitution  Phlegm-dampness constitution  Dampness-heat constitution  Blood stasis constitution  Qi stagnation constitution  Inherited special constitution | 263 (64.9)  38 (9.3)  23 (5.6)  28 (6.9)  15 (3.7)  15 (3.7)  0 (0)  21 (5.1)  2 (0.4) | 84 (10.2)  152 (18.5)  201 (24.5)  44 (5.3)  132 (16.1)  102 (12.4)  19 (2.3)  76 (9.2)  8 (0.9) | 413.9 | 0.000 |

**Supplementary Table 27 | Univariate analysis of TCM constitution type between PLGC patients and GC patients [n (%)].**

| **Factor** | **Category** | **PLGC**  **(n=1006)** | **GC**  **(n=818)** | **χ^2^** | ***P*** |
| --- | --- | --- | --- | --- | --- |
| TCM constitution type | Balanced constitution  Qi-deficiency constitution  Yang-deficiency constitution  Yin-deficiency constitution  Phlegm-dampness constitution  Dampness-heat constitution  Blood stasis constitution  Qi stagnation constitution  Inherited special constitution | 146 (14.5)  257 (25.5)  129 (12.8)  78 (7.7)  115 (11.4)  87 (8.6)  39 (3.8)  144 (14.3)  11 (1.0) | 84 (10.2)  152 (18.5)  201 (24.5)  44 (5.3)  132 (16.1)  102 (12.4)  19 (2.3)  76 (9.2)  8 (0.9) | 93.88 | 0.000 |

**Supplementary Table 28 | Logistic regression analysis of risk factors between healthy people and PLGC patients.**

| **Factor** | ***β*** | ***SE*** | ***Wald*χ*^2^*** | ***P*** | **OR** | **95%CI** | |
| --- | --- | --- | --- | --- | --- | --- | --- |
|  |  |  |  |  |  | **Lower bound** | **Upper bound** |
| Age(years) (≤30) |  |  | 219.069 | 0.000 |  |  |  |
| 31 ~ 40 | 2.900 | 0.230 | 159.118 | 0.000 | 18.172 | 11.58 | 28.516 |
| 41 ~ 50 | 4.034 | 0.369 | 119.585 | 0.000 | 56.469 | 27.40 | 116.35 |
| >50 | 3.651 | 0.401 | 83.094 | 0.000 | 38.525 | 17.571 | 84.470 |
| BMI (<18.5) |  |  | 26.105 | 0.000 |  |  |  |
| 18.5 ~ 24.0 | -1.721 | 0.380 | 20.470 | 0.000 | 0.179 | 0.085 | 0.377 |
| >24.0 | -0.554 | 0.541 | 1.049 | 0.306 | 0.574 | 0.199 | 1.660 |
| Alcohol consumption history (no) |  |  | 4.541 | 0.209 |  |  |  |
| <5 years | 0.382 | 0.266 | 2.065 | 0.151 | 1.465 | 0.870 | 2.468 |
| 5 ~ 10 years | 0.388 | 0.306 | 1.602 | 0.206 | 1.474 | 0.808 | 2.687 |
| >10 years | 0.599 | 0.397 | 2.271 | 0.132 | 1.820 | 0.835 | 3.966 |
| Hypertension | 0.243 | 0.284 | 0.730 | 0.393 | 1.275 | 0.730 | 2.226 |
| Family history | 0.501 | 0.272 | 3.379 | 0.066 | 1.650 | 0.967 | 2.815 |
| Irregular sleep | 0.347 | 0.288 | 1.459 | 0.227 | 1.415 | 0.806 | 2.487 |
| Sports situation (Less) |  |  | 17.39 | 0.001 |  |  |  |
| Everyday | -0.703 | 0.291 | 5.828 | 0.016 | 0.495 | 0.280 | 0.876 |
| Every 3-5 days | -0.887 | 0.294 | 9.138 | 0.003 | 0.412 | 0.232 | 0.732 |
| Once a week | -0.929 | 0.250 | 13.82 | 0.000 | 0.395 | 0.242 | 0.644 |
| Pickled food (Never) |  |  | 22.03 | 0.000 |  |  |  |
| Sometimes | 1.001 | 0.305 | 10.81 | 0.001 | 2.722 | 1.499 | 4.945 |
| Often | 1.481 | 0.401 | 13.65 | 0.000 | 4.398 | 2.004 | 9.647 |
| Overhot food (Never) |  |  | 3.462 | 0.177 |  |  |  |
| Sometimes | 0.222 | 0.314 | 0.500 | 0.479 | 1.249 | 0.675 | 2.310 |
| Often | 0.641 | 0.357 | 3.215 | 0.073 | 1.898 | 0.942 | 3.825 |
| Irregular meals (Never) |  |  | 3.198 | 0.202 |  |  |  |
| Sometimes | 0.089 | 0.294 | 0.092 | 0.761 | 1.093 | 0.615 | 1.946 |
| Often | 0.748 | 0.420 | 3.180 | 0.075 | 2.113 | 0.929 | 4.810 |
| Tongue color (Light red tongue) |  |  | 27.69 | 0.000 |  |  |  |
| Pale tongue | 1.041 | 0.238 | 19.16 | 0.000 | 2.832 | 1.777 | 4.513 |
| Crimson tongue | 1.418 | 0.398 | 12.66 | 0.000 | 4.127 | 1.890 | 9.009 |
| Livid tongue | 20.627 | 3924.3 | 0.000 | 0.996 | 908616954.8 | 0.000 | . |
| Tongue coating color (White tongue fur) |  |  | 8.110 | 0.044 |  |  |  |
| Yellow tongue fur | 0.736 | 0.258 | 8.110 | 0.004 | 2.087 | 1.258 | 3.462 |
| Grey tongue fur | 19.757 | 4254.6 | 0.000 | 0.996 | 380689200.88 | 0.000 | . |
| Black tongue fur | 22.066 | 11463.845 | 0.000 | 0.998 | 3831210662.918 | 0.000 | . |
| Thin or thick coating of the tongue | 0.483 | 0.246 | 3.854 | 0.050 | 1.621 | 1.001 | 2.626 |
| Greasy fur | 0.330 | 0.293 | 1.270 | 0.260 | 1.391 | 0.784 | 2.470 |
| Curdy fur | 0.310 | 0.329 | 0.887 | 0.346 | 1.363 | 0.715 | 2.597 |
| TCM constitution type (Balanced constitution) |  |  | 126.9 | 0.000 |  |  |  |
| Qi-deficiency constitution | 2.299 | 0.286 | 64.56 | 0.000 | 9.964 | 5.687 | 17.458 |
| Yang-deficiency constitution | 1.987 | 0.350 | 32.16 | 0.000 | 7.293 | 3.670 | 14.493 |
| Yin-deficiency constitution | 1.975 | 0.399 | 24.44 | 0.000 | 7.206 | 3.294 | 15.763 |
| Phlegm-dampness constitution | 2.483 | 0.409 | 36.93 | 0.000 | 11.981 | 5.379 | 26.687 |
| Dampness-heat constitution | 2.093 | 0.416 | 25.33 | 0.000 | 8.109 | 3.589 | 18.319 |
| Blood stasis constitution | 20.339 | 5818.020 | 0.000 | 0.997 | 681228403.5 | 0.000 | . |
| Qi stagnation constitution | 3.044 | 0.364 | 69.95 | 0.000 | 20.997 | 10.28 | 42.856 |
| Inherited special constitution | 2.136 | 1.030 | 4.301 | 0.038 | 8.468 | 1.124 | 63.776 |
| Constant | -2.116 | 0.454 | 21.67 | 0.000 | 0.121 |  |  |

**Supplementary Table 29 | Logistic regression analysis of risk factors between healthy people and GC patients.**

| **Factor** | ***β*** | ***SE*** | ***Wald*χ*^2^*** | ***P*** | **OR** | **95%CI** | |
| --- | --- | --- | --- | --- | --- | --- | --- |
|  |  |  |  |  |  | **Lower bound** | **Upper bound** |
| Age (years) (≤30) |  |  | 65.088 | 0.000 |  |  |  |
| 31 ~ 40 | 4.303 | 0.825 | 27.238 | 0.000 | 73.942 | 14.691 | 372.17 |
| 41 ~ 50 | 8.264 | 1.205 | 47.022 | 0.000 | 38.989 | 365.710 | 41185.892 |
| >50 | 10.043 | 1.256 | 63.914 | 0.000 | 229.527 | 1960.271 | 269708.814 |
| BMI (<18.5) |  |  | 13.301 | 0.001 |  |  |  |
| 18.5 ~ 24.0 | -3.229 | 0.906 | 12.711 | 0.000 | 0.040 | 0.007 | 0.234 |
| >24.0 | -3.824 | 1.325 | 8.327 | 0.004 | 0.022 | 0.002 | 0.293 |
| Alcohol consumption history (no) |  |  | 9.162 | 0.027 |  |  |  |
| <5 years | 0.760 | 0.613 | 1.536 | 0.215 | 2.138 | 0.643 | 7.110 |
| 5 ~ 10 years | 1.015 | 0.682 | 2.216 | 0.137 | 2.760 | 0.725 | 10.502 |
| >10 years | 2.808 | 0.977 | 8.264 | 0.004 | 16.571 | 2.443 | 112.38 |
| Family history | 1.841 | 0.674 | 7.463 | 0.006 | 6.302 | 1.682 | 23.604 |
| Anaemia | -0.672 | 0.705 | 0.908 | 0.341 | 0.511 | 0.128 | 2.034 |
| Sleep onset latency (<30min) |  |  | 7.917 | 0.019 |  |  |  |
| 30 ~ 60min | 1.730 | 0.657 | 6.923 | 0.009 | 5.639 | 1.555 | 20.455 |
| >60min | 1.090 | 0.835 | 1.705 | 0.192 | 2.975 | 0.579 | 15.286 |
| Chronic sleep deprivation | 1.972 | 0.732 | 7.267 | 0.007 | 7.189 | 1.713 | 30.162 |
| Irregular sleep | 1.585 | 0.913 | 3.014 | 0.083 | 4.879 | 0.815 | 29.201 |
| Anxiety (Never) |  |  | 7.333 | 0.026 |  |  |  |
| Minor | 1.854 | 0.721 | 6.609 | 0.010 | 6.387 | 1.554 | 26.255 |
| Severe | 2.447 | 2.265 | 1.167 | 0.280 | 11.559 | 0.136 | 980.099 |
| Sports situation (Less) |  |  | 8.334 | 0.040 |  |  |  |
| Everyday | 0.973 | 0.722 | 1.816 | 0.178 | 2.645 | 0.643 | 10.888 |
| Every 3-5 days | -0.620 | 0.699 | 0.789 | 0.374 | 0.538 | 0.137 | 2.115 |
| Once a week | -1.172 | 0.674 | 3.021 | 0.082 | 0.310 | 0.083 | 1.161 |
| Pickled food (Never) |  |  | 16.221 | 0.000 |  |  |  |
| Sometimes | 2.169 | 0.748 | 8.417 | 0.004 | 8.752 | 2.021 | 37.894 |
| Often | 3.309 | 0.958 | 11.930 | 0.001 | 27.353 | 4.184 | 178.833 |
| Overhot food (Never) |  |  | 5.829 | 0.054 |  |  |  |
| Sometimes | 0.784 | 0.610 | 1.654 | 0.198 | 2.191 | 0.663 | 7.237 |
| Often | 2.325 | 1.019 | 5.204 | 0.023 | 10.227 | 1.388 | 75.383 |
| Irregular meals (Never) |  |  | 14.540 | 0.001 |  |  |  |
| Sometimes | 2.773 | 0.729 | 14.488 | 0.000 | 16.009 | 3.839 | 66.762 |
| Often | 0.649 | 0.953 | 0.463 | 0.496 | 1.913 | 0.295 | 12.388 |
| Tongue color (Light red tongue) |  |  | 15.889 | 0.001 |  |  |  |
| Pale tongue | 1.784 | 0.564 | 9.999 | 0.002 | 5.952 | 1.970 | 17.982 |
| Crimson tongue | 2.617 | 0.842 | 9.669 | 0.002 | 13.698 | 2.632 | 71.303 |
| Livid tongue | 17.888 | 3964.900 | 0.000 | 0.996 | 58720191.889 | 0.000 | . |
| Tongue coating color (White tongue fur) |  |  | 12.400 | 0.006 |  |  |  |
| Yellow tongue fur | 2.011 | 0.571 | 12.400 | 0.000 | 7.472 | 2.439 | 22.886 |
| Grey tongue fur | 18.654 | 3276.053 | 0.000 | 0.995 | 126288799.418 | 0.000 | . |
| Black tongue fur | 22.226 | 6622.153 | 0.000 | 0.997 | 4496150562.56 | 0.000 | . |
| Thin or thick coating of the tongue | 1.430 | 0.557 | 6.604 | 0.010 | 4.180 | 1.404 | 12.445 |
| Greasy fur | 1.285 | 0.666 | 3.724 | 0.054 | 3.616 | 0.980 | 13.339 |
| TCM constitution type (Balanced constitution) |  |  | 33.712 | 0.000 |  |  |  |
| Qi-deficiency constitution | 2.230 | 0.746 | 8.928 | 0.003 | 9.300 | 2.154 | 40.159 |
| Yang-deficiency constitution | 4.367 | 1.008 | 18.765 | 0.000 | 78.785 | 10.924 | 568.203 |
| Yin-deficiency constitution | 3.732 | 1.188 | 9.870 | 0.002 | 41.767 | 4.071 | 428.549 |
| Phlegm-dampness constitution | 4.901 | 1.071 | 20.917 | 0.000 | 134.360 | 16.452 | 1097.31 |
| Dampness-heat constitution | 3.167 | 0.902 | 12.317 | 0.000 | 23.743 | 4.049 | 139.225 |
| Blood stasis constitution | 24.654 | 6280.78 | 0.000 | 0.997 | 50948751353.1 | 0.000 | . |
| Qi stagnation constitution | 4.878 | 1.091 | 19.974 | 0.000 | 131.360 | 15.468 | 1115.59 |
| Inherited special constitution | 4.420 | 1.719 | 6.610 | 0.010 | 83.093 | 2.859 | 2414.96 |
| Constant | -10.664 | 1.660 | 41.288 | 0.000 | 0.000 |  |  |

**Supplementary Table 30 | Analysis of binary Logistic regression model for PLGC and GC patients.**

| **Focter** | ***β*** | ***SE*** | ***Wald*χ*^2^*** | ***P*** | **OR** | **95%CI** | |
| --- | --- | --- | --- | --- | --- | --- | --- |
|  |  |  |  |  |  | **Lower bound** | **Upper bound** |
| Age(years) (≤30) |  |  | 418.146 | 0.000 |  |  |  |
| 31 ~ 40 | 0.512 | 0.363 | 1.985 | 0.159 | 1.669 | 0.819 | 3.402 |
| 41 ~ 50 | 1.791 | 0.359 | 24.880 | 0.000 | 5.995 | 2.966 | 12.119 |
| >50 | 3.885 | 0.360 | 116.759 | 0.000 | 48.691 | 24.064 | 98.520 |
| BMI (<18.5) |  |  | 23.387 | 0.000 |  |  |  |
| 18.5 ~ 24.0 | -0.723 | 0.155 | 21.849 | 0.000 | 0.485 | 0.358 | 0.657 |
| >24.0 | -0.816 | 0.265 | 9.505 | 0.002 | 0.442 | 0.263 | 0.743 |
| Alcohol consumption history (no) |  |  | 33.103 | 0.000 |  |  |  |
| <5 years | 0.878 | 0.179 | 23.955 | 0.000 | 2.406 | 1.693 | 3.420 |
| 5 ~ 10 years | 0.552 | 0.178 | 9.631 | 0.002 | 1.736 | 1.225 | 2.459 |
| >10 years | 0.850 | 0.219 | 15.036 | 0.000 | 2.339 | 1.522 | 3.593 |
| Family history | 0.343 | 0.149 | 5.309 | 0.021 | 1.409 | 1.053 | 1.886 |
| Sleep onset latency (<30min) |  |  | 7.558 | 0.023 |  |  |  |
| 30 ~ 60min | 0.424 | 0.165 | 6.582 | 0.010 | 1.528 | 1.105 | 2.112 |
| >60min | 0.320 | 0.219 | 2.132 | 0.144 | 1.377 | 0.896 | 2.116 |
| Chronic sleep deprivation | 0.709 | 0.182 | 15.171 | 0.000 | 2.032 | 1.422 | 2.903 |
| Anxiety (Never) |  |  | 9.035 | 0.011 |  |  |  |
| Minor | 0.567 | 0.192 | 8.747 | 0.003 | 1.762 | 1.211 | 2.565 |
| Severe | 0.225 | 0.253 | .795 | 0.373 | 1.253 | 0.763 | 2.056 |
| Sports situation (Less) |  |  | 34.599 | 0.000 |  |  |  |
| Everyday | 0.197 | 0.172 | 1.318 | 0.251 | 1.218 | 0.870 | 1.704 |
| Every 3-5 days | -0.723 | 0.195 | 13.674 | 0.000 | 0.485 | 0.331 | 0.712 |
| Once a week | -0.775 | 0.192 | 16.308 | 0.000 | 0.461 | 0.316 | 0.671 |
| Pickled food (Never) |  |  | 24.223 | 0.000 |  |  |  |
| Sometimes | 0.335 | 0.158 | 4.474 | 0.034 | 1.398 | 1.025 | 1.907 |
| Often | 0.924 | 0.191 | 23.436 | 0.000 | 2.519 | 1.733 | 3.662 |
| Overhot food (Never) |  |  | 27.983 | 0.000 |  |  |  |
| Sometimes | 0.611 | 0.175 | 12.202 | 0.000 | 1.842 | 1.307 | 2.594 |
| Often | 0.936 | 0.206 | 20.576 | 0.000 | 2.549 | 1.701 | 3.820 |
| Tongue color (Light red tongue) |  |  | 32.190 | 0.000 |  |  |  |
| Pale tongue | 0.797 | 0.157 | 25.777 | 0.000 | 2.219 | 1.631 | 3.019 |
| Crimson tongue | 0.812 | 0.188 | 18.654 | 0.000 | 2.252 | 1.558 | 3.255 |
| Livid tongue | 0.594 | 0.260 | 5.211 | 0.022 | 1.811 | 1.088 | 3.017 |
| Tongue coating color (White tongue fur) |  |  | 13.582 | 0.004 |  |  |  |
| Yellow tongue fur | 0.256 | 0.147 | 3.046 | 0.081 | 1.291 | 0.969 | 1.721 |
| Grey tongue fur | 0.645 | 0.253 | 6.508 | 0.011 | 1.905 | 1.161 | 3.126 |
| Black tongue fur | 1.462 | 0.570 | 6.588 | 0.010 | 4.314 | 1.413 | 13.174 |
| Thin or thick coating of the tongue | 0.323 | 0.142 | 5.158 | 0.023 | 1.381 | 1.045 | 1.825 |
| Greasy fur | 0.379 | 0.162 | 5.493 | 0.019 | 1.461 | 1.064 | 2.006 |
| TCM constitution type (Balanced constitution) |  |  | 46.753 | 0.000 |  |  |  |
| Qi-deficiency constitution | 0.014 | 0.237 | 0.004 | 0.952 | 1.014 | 0.638 | 1.613 |
| Yang-deficiency constitution | 0.936 | 0.245 | 14.591 | 0.000 | 2.550 | 1.577 | 4.123 |
| Yin-deficiency constitution | -0.122 | 0.325 | 0.142 | 0.706 | 0.885 | 0.468 | 1.672 |
| Phlegm-dampness constitution | 0.864 | 0.263 | 10.772 | 0.001 | 2.374 | 1.417 | 3.977 |
| Dampness-heat constitution | 0.930 | 0.277 | 11.237 | 0.001 | 2.535 | 1.472 | 4.366 |
| Blood stasis constitution | -0.105 | 0.433 | 0.059 | 0.808 | 0.900 | 0.385 | 2.104 |
| Qi stagnation constitution | 0.017 | 0.276 | 0.004 | 0.951 | 1.017 | 0.592 | 1.748 |
| Inherited special constitution | 0.777 | 0.705 | 1.215 | 0.270 | 2.175 | 0.546 | 8.660 |
| Constant | -4.129 | 0.437 | 89.260 | 0.000 | 0.016 |  |  |
